# Supramolecular Motifs in the Crystal Structures of Triethylbenzene Derivatives Bearing Pyridinium Subunits in Combination with Pyrimidinyl or Pyridinyl Groups

**DOI:** 10.3390/molecules28186485

**Published:** 2023-09-07

**Authors:** Andrea Weiße, Wilhelm Seichter, Monika Mazik

**Affiliations:** Institut für Organische Chemie, Technische Universität Bergakademie Freiberg, Leipziger Straße 29, 09596 Freiberg, Germany; andrea.weisse@extern.tu-freiberg.de (A.W.); wilhelm.seichter@web.de (W.S.)

**Keywords:** molecular recognition, anion receptors, supramolecular motifs, counterion

## Abstract

A series of mono- and dicationic 1,3,5-trisubstituted 2,4,6-triethylbenzenes containing pyridinium groups in combination with aminopyrimidine-/aminopyridine-based recognition units were synthesized and crystallographically studied. The combination of neutral and ionic building blocks represents a promising strategy for the development of effective and selective artificial receptors for anionic substrates. In the crystalline state, the investigated compounds show a tendency to bind the counterion PF_6_^−^ in the cavity formed by the three functionalized side-arms. The intermolecular interactions with the PF_6_^−^ ion comprise N-H∙∙∙F and C-H∙∙∙F bonds. Detailed analysis of various supramolecular motifs, including interactions with solvent molecules, provides deeper insights into the processes of molecular recognition. The information obtained is useful in the development of new receptor molecules for anions and in the selection of the most appropriate counterion.

## 1. Introduction

The extensive studies performed by our group to develop artificial carbohydrate receptors have clearly demonstrated that the consideration of different receptor building blocks capable of forming multiple noncovalent interactions is a promising strategy to achieve the effective and selective recognition of carbohydrates [1,2,3,4,5,6,7,8]. The use of such a recognition strategy, based on combined noncovalent interactions, is known from carbohydrate-binding proteins [9,10] and served as a source of inspiration for studies with artificial systems.

In the development of new receptor molecules, the combination of different neutral functional groups, as well as of neutral and ionic residues, has been considered. As examples of receptors bearing both neutral and ionic recognition groups, the representatives of 1,3,5-substituted 2,4,6-triethylbenzene derivatives can be mentioned, [5] which were developed for the recognition of the anionic carbohydrate *N*-acetylneuraminic acid (Neu5Ac), the most abundant sialic acid (for a discussion on the importance of the detection of free sialic acids in biological samples, see [11]). These receptor molecules contain cationic pyridinium/quinolinium groups in combination with neutral moieties, such as aminopyridine-based recognition groups, and are able to complex Neu5Ac in highly competitive aqueous media. The building blocks of these receptor molecules enable Neu5Ac binding through a combination of neutral/charge-reinforced hydrogen bonds, ion pairs, CH-π interactions and van der Waals forces. In contrast, the identically substituted triethylbenzene derivatives with three cationic recognition sites are unable to effectively complex Neu5Ac in aqueous media but showed strong binding in less competitive solvents, such as acetonitrile, as expected. At this point, it should be mentioned that studies with triethylbenzene-based compounds possessing three cationic groups, such as pyridinium, bipyridinium, quinolinium or imidazolium groups, have made a very important contribution to the field of anion recognition [12,13,14,15,16,17,18,19,20,21,22,23,24,25,26,27].

In this work, we report the syntheses and crystal structures of new representatives of 1,3,5-substituted 2,4,6-triethylbenzene derivatives containing pyridinium groups (3-methylpyridinium, 3-hydroxypyridinium or 2-amino-5-methylpyridinium) in combination with aminopyrimidine-based recognition moieties. These new compounds are analogues of the aforementioned Neu5Ac receptor molecules with aminopyridine-based recognition groups. Their molecular architecture offers numerous possibilities for structural variations, enabling the synthesis of a whole range of compounds for systematic binding studies. It should be noted that recognition strategies for anionic substrates based on combined noncovalent interactions have been very well summarized in a review article [28] (for examples of reviews on anion recognition, see refs. [29,30,31,32,33,34]).

**Figure 1:**
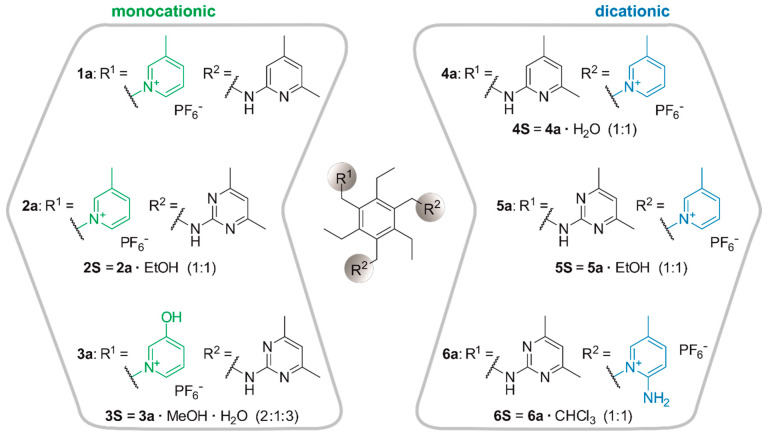
Structures of the PF_6_^−^ salts **1a**–**6a** and the composition of the solvates **2S**–**6S**.

Here, the crystal structures of salts of mono- and dicationic triethylbenzene derivatives **1a**–**6a** with PF_6_^−^ as counterions are described, comprising the solvent-free crystal structure of **1a** and the solvates **2a·**EtOH (**2S**), **3a·**MeOH**·**H_2_O (2:1:3; **3S**), **4a·**H_2_O (**4S**), **5a·**EtOH (**5S**) and **6a·**CHCl_3_ (**6S**) (see Figure 1). In addition to the crystal structures of the new compounds **2a**, **3a**, **5a** and **6a** with pyrimidinyl groups, the crystal structures of two compounds containing pyridinyl groups (compounds **1a** and **4a**) are also considered for comparative purposes.

Detailed analysis of the various supramolecular motifs observed in the crystal structures of the hexafluorophosphate salts **1a**–**6a**, including motifs involving solvent molecules, provides interesting insights into molecular recognition phenomena, making the obtained results valuable, among others, for the development of new artificial receptor molecules.

## 2. Results and Discussion

### 2.1. Synthesis

**Scheme 1:**
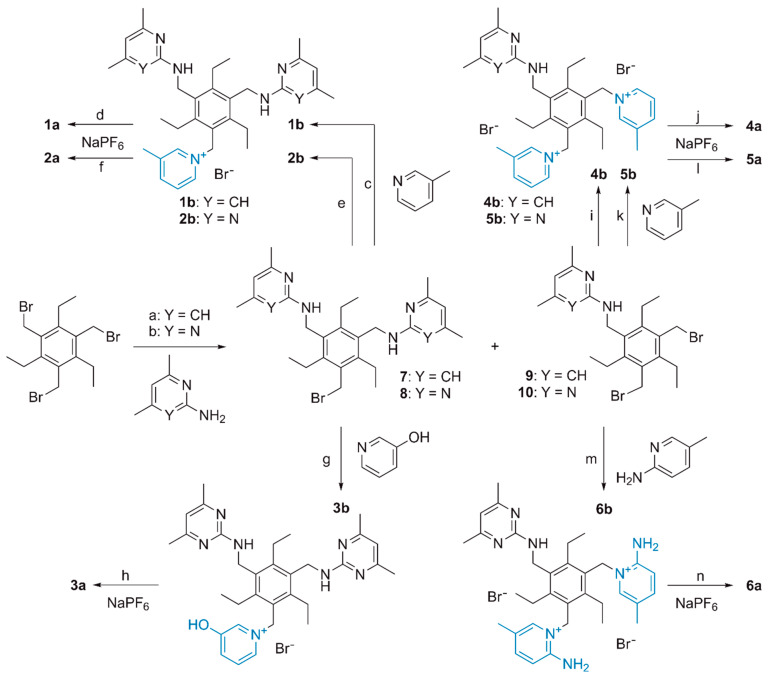
Synthesis of the bromide salts **1b**–**6b** and the hexafluorophosphate salts **1a**–**6a**. Reagents and conditions: (a) CH_3_CN/THF, K_2_CO_3_, rt [35]; (b) CH_3_CN/THF, K_2_CO_3_, 50 °C [36]; (c) CHCl_3_, 2 d, reflux (40% of **1b**); (d) H_2_O/MeOH, NaPF_6_, 2 d, rt (62% of **1a**); (e) THF, 8 h, reflux/4 h, rt (70% of **2b**); (f) MeOH/EtOH, NaPF_6_, 1 d, rt (53% of **2a**); (g) toluene/MeOH, 8 h, reflux (92% of **3b**); (h) H_2_O/MeOH, NaPF_6_, 2 h, 50 °C/4 h, rt (82% of **3a**); (i) acetone, 2 h, reflux (60% of **4b**); (j) MeOH, NaPF_6_, 2 d, rt (92% of **4a**); (k) CH_2_Cl_2_, 8 h, reflux/4 h, rt (86% of **5b**); (l) MeOH, NaPF_6_, 1 d, rt (63% of **5a**); (m) CH_2_Cl_2_, 15 h, reflux/4 h, rt (62% of **6b**); (n) H_2_O, NaPF_6_, 3 h, 40 °C/4 h, rt (63% of **6a**).

The syntheses of the bromide salts **1b**–**6b** and the corresponding hexafluorophosphates **1a**–**6a** are summarized in Scheme 1 (see Section 4 for details). The bromide salts **1b**–**3b**, containing one pyridinium group (monocationic compounds), were prepared by the reaction of 1-bromomethyl-3,5-bis[(4,6-dimethylpyridin-2-yl)aminomethyl]-2,4,6-triethy-lbenzene (**7**) or 1-bromomethyl-3,5-bis[(4,6-dimethylpyrimidin-2-yl)aminomethyl]-2,4,6-triethyl-benzene (**8**) with 3-methylpyridine (in the cases of **1b** and **2b**) or 3-hydroxypyridine (in the case of **3b**). For the synthesis of the bromide salts **4b**–**6b**, bearing two pyridinium groups (dicationic derivatives), 1,3-bis(bromomethyl)-5-(4,6-dimethylpyridin-2-yl)aminomethyl-2,4,6-triethylbenzene (**9**) and 1,3-bis(bromomethyl)-5-(4,6-dimethylpyrimidin-2-yl)aminomethyl-2,4,6-triethylbenzene (**10**) acted as starting materials. The treatment of **9** with 3-methylpyridine gave compound **4b**, while the reaction of **10** with 3-methylpyridine or 2-amino-5-methylpyridine provided **5b** and **6b**, respectively.

Starting materials **7**–**10** can be obtained by the reaction of 1,3,5-tris(bromomethyl)-2,4,6-triethylbenzene with 2-amino-4,6-dimethylpyridine or 2-amino-4,6-dimethylpyrimidine in the presence of potassium carbonate as a base, as reported by us previously [35,36].

The bromide salts were converted to the corresponding hexafluorophosphates **1a**–**6a** by treatment with an excess of sodium hexafluorophosphate in water or methanol.

### 2.2. Crystallographic Studies

**Figure 2:**
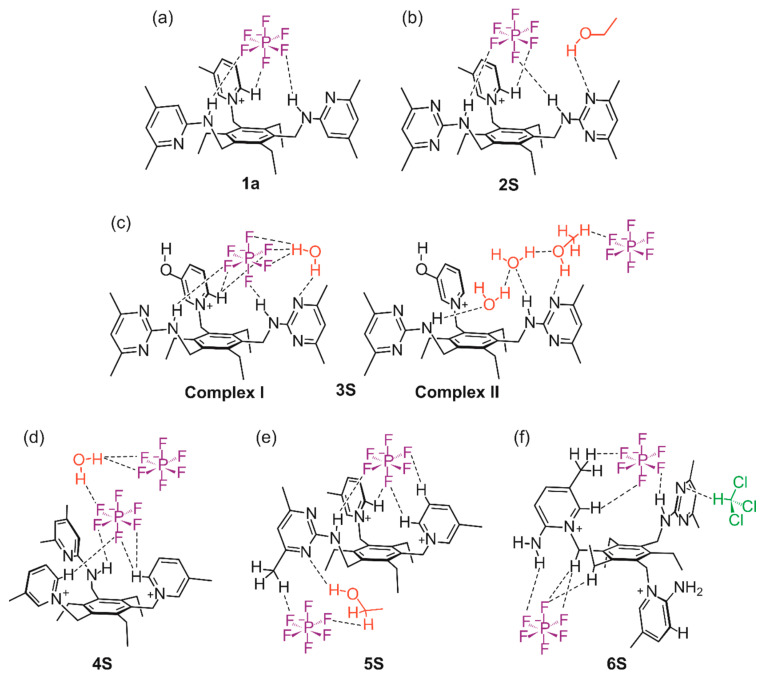
Schematic illustration of **1^+^**PF_6_^−^ (**a**), **2^+^**PF_6_^−^**·**EtOH (**b**), **3^+^**PF_6_^−^**·**H_2_O (complex I) and **3^+^**PF_6_^−^**·**(H_2_O)_2_**·**CH_3_OH (complex II) (**c**), **4**^2**+**^(PF_6_^−^)_2_**·**H_2_O (**d**), **5**^2**+**^(PF_6_^−^)_2_**·**EtOH (**e**) and **6**^2**+**^(PF_6_^−^)_2_**·**CHCl_3_ (**f**).

Crystals suitable for X-ray diffraction analysis were grown by the slow evaporation of the solvent from solutions of the respective compounds. In the cases of **1a**, **3a**, **4a** and **6a**, the corresponding compound was suspended in water and mixed with small amounts of methanol, chloroform or acetonitrile until a clear solution was formed. Crystal growth then occurred by the slow evaporation of the organic solvent, yielding the solvent-free structure **1a** (see Figure 2) as well as the solvates **3a·**MeOH**·**H_2_O (2:1:3; **3S**), **4a·**H_2_O (**4S**) and **6a·**CHCl_3_ (**6S**). Crystals of compounds **2a** and **5a** were obtained from ethanol as solvates **2a·**EtOH (**2S**) and **5a·**EtOH (**5S**), respectively. In the case of **3S**, two complexes **3S-I** (**3^+^**PF_6_^−^**·**H_2_O) and **3S-II** [**3^+^**PF_6_^−^**·**(H_2_O)_2_**·**CH_3_OH] were observed, as shown in Figure 2.

Crystallographic data and the selected refinement parameters of the crystal structures are provided in Table S1. The geometric features of the receptors are described by the calculation of dihedral angles between the aromatic rings, designated as A–D in the figures showing the molecular structures. Their values, together with relevant torsion angles, are presented in Table S2, while information regarding the non-covalent molecular interactions in the crystals is given in Table S3.

#### 2.2.1. Hexafluorophosphate Salts of the Monocationic Tripodal Receptors: Solvent-Free Structure **1a** and Solvates **2S** and **3S**

In the crystal structures of **1a** (**1^+^**PF_6_^−^), **2S** (**2^+^**PF_6_^−^**·**EtOH) and **3S** (**3^+^**PF_6_^−^**·**MeOH**·**H_2_O; 2:1:3), the receptor cations reveal common structural features showing an alternating arrangement of the substituents above and below the plane of the central arene ring, which is the frequently observed conformation of molecules based on the 1,3,5-triethylbenzene platform [37,38,39]. Taking the ethyl groups into account, the spatial arrangement of the substituents along the periphery of the benzene ring represents an *ab′ab′ab′* pattern (*a* = above, *b* = below, *a′*/*b′* = ethyl; for details, see references [40,41]).

**Figure 3:**
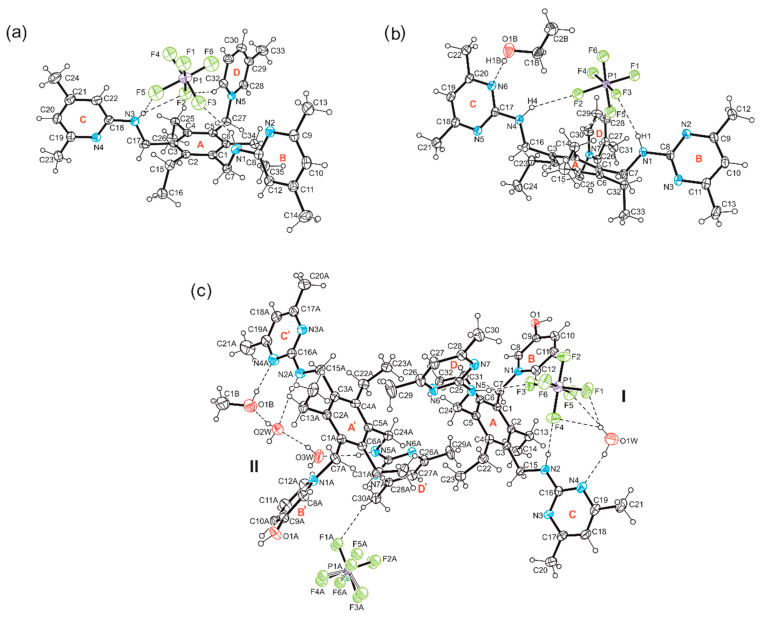
Perspective views of the molecular structures of **1^+^**PF_6_^−^ (**1a**) (**a**), **2^+^**PF_6_^−^**·**EtOH (**2S**) (**b**) and **3^+^**PF_6_^−^**·**MeOH**·**H_2_O [2:1:3; **3S**] (**c**), including atom labeling and ring specification. Displacement ellipsoids are drawn at the 50% probability level. Dashed lines represent hydrogen bond interactions.

**Figure 4:**
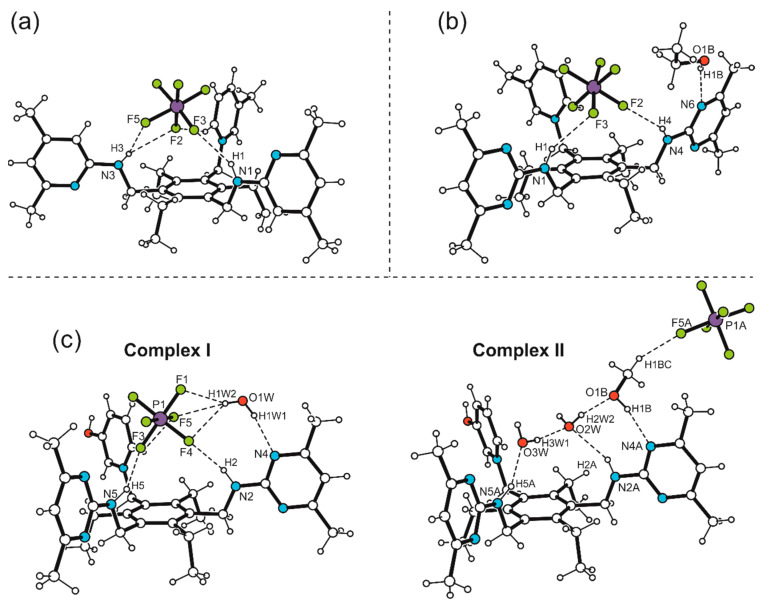
Ball-and-stick representations (side views) of the molecular structures of **1a** (**a**), **2a·**EtOH (**2S**) (**b**) and **3a·**MeOH**·**H_2_O (2:1:3; **3S**) (**c**), including the labeling of coordinating atoms. For the sake of clarity, only one component of the disordered anions is shown.

In the solvent-free structure **1a** (space group *P*-1, *Z* = 2), which is illustrated in Figure 2a, Figure 3a and Figure 4a, the PF_6_^−^ ion resides in the receptor cavity created by the functionalized side-arms bearing heterocyclic groups. The two pyridinylaminomethyl moieties of the receptor display a significant twist, which is reflected by torsion angles of −156.5(3) and −170.3(3)° for their atomic sequences C_benzene_-C-N-C_pyridine_. The pyridine rings (B, C) of these substituents are inclined at angles of 75.5(1) and 81.8(1)° with respect to the plane of the central arene ring (A), whereas the pyridinium group (D) is oriented nearly perpendicular (89.5(1)°) to this plane. The interactions between the receptor cation and the counter ion comprise N-H∙∙∙F bonds [*d*(H∙∙∙F) 2.29(6)–2.58(4) Å] and a relatively short C-H∙∙∙F bond [*d*(H···F) 2.37 Å], the latter involving an *ortho*-H atom of the pyridinium group. It should be mentioned that the participation of the fluorine atom of PF_6_^–^ in the formation of hydrogen bonds with a variety of X-H donors [X = C, N, O.; X-H···F(P) interaction] was discussed in reference [42] (for a review on hydrogen bonds with organic fluorine, including C-H···F(C), N-H···F(C) and O-H···F(C) bonds, see reference [43]).

**Figure 5:**
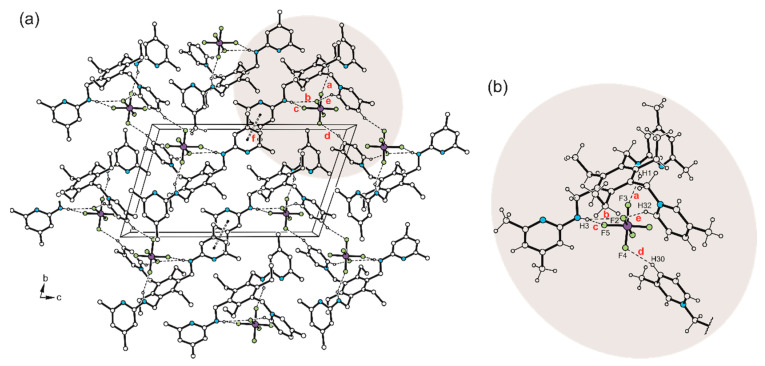
Packing diagram of **1^+^**PF_6_^−^ viewed down the crystallographic *a*-axis (**a**) with an excerpt (grey) highlighting the interactions between the receptor cation and the PF_6_^−^ anion (**b**). For the sake of clarity, all hydrogen atoms excluded from hydrogen bonding are omitted in (**a**). Nitrogen atoms are displayed as blue, phosphorus as violet and fluorine atoms as green circles. Dashed lines represent hydrogen bonds, dashed double lines represent *π*∙∙∙*π* arene interactions. The following interactions are shown: N-H∙∙∙F (a–c = 2.29–2.58 Å); C-H∙∙∙F (d,e = 2.37, 2.36 Å); *π*∙∙∙*π* (f = 3.86 Å).

As is obvious from Figure 5, the crystal of **1a** is composed of dimers, in which two ion pairs **1^+^**PF_6_^−^ are connected by C-H∙∙∙F bonds involving the *para*-H atom of the pyridinium ring. These dimers are further linked by offset *π*∙∙∙*π* interactions [44,45] with a *centroid*–*centroid* distance of 3.862(2) Å between the interacting pyridine rings.

**Figure 6:**
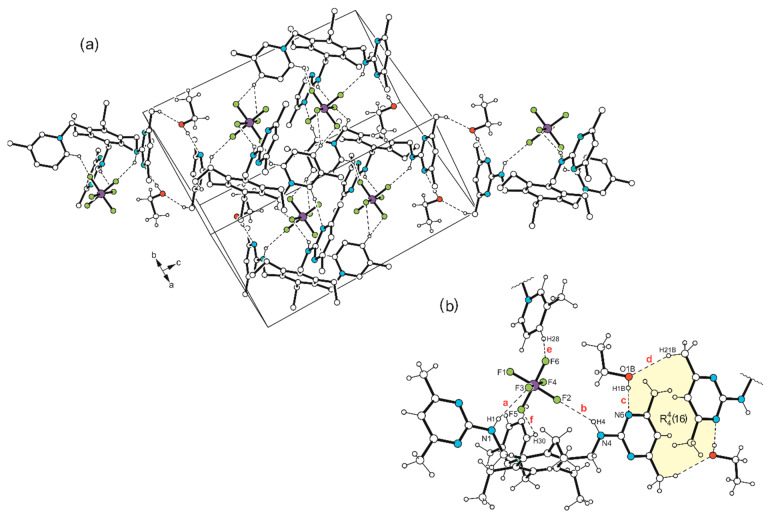
(**a**) Excerpt of the crystal structure of the complex **2a·**EtOH (**2S**) and (**b**) structure motif including the labeling of coordinating atoms. Only the major disorder position of the PF_6_^−^ ion is shown for clarity. Dashed lines represent hydrogen bond interactions. In (**b**), the 16-membered ring motif is marked by color highlighting. Nitrogen atoms are displayed as blue, oxygen as red, phosphorus as violet and fluorine atoms as green circles. In (**b**), the following interactions are shown: N-H∙∙∙F (a/b = 2.30, 2.46 Å), O-H∙∙∙N (c = 2.02 Å), C-H∙∙∙O (d = 2.57 Å) and C-H∙∙∙F (e/f = 2.35, 2.51 Å).

Hexafluorophosphate salt **2a** crystallizes from ethanol as colorless rods of the space group *P*-1 with the asymmetric part of the unit cell containing one receptor cation, one PF_6_^−^ ion disordered over three positions and one solvent molecule (solvate **2S**). The ORTEP diagram and a ball-and-stick representation of the complex **2^+^**PF_6_**^−^·**EtOH are shown in Figure 3b and Figure 4b, respectively. The structure of the receptor-anion unit **2^+^**PF_6_^−^ is similar to that of **1^+^**PF_6_^−^. Also, in the present case, the PF_6_^−^ ion is connected to the receptor by N-H∙∙∙F bonds [*d*(H∙∙∙F) 2.30(2)–2.52(2) Å] and a C-H∙∙∙F interaction [*d*(H∙∙∙F) 2.51 Å]. The alcohol molecule is associated via O-H∙∙∙N bonding to the pyrimidine nitrogen N6 [*d*(H∙∙∙N) 2.02(2) Å]. As displayed in Figure 6, C_pyridinium_-H∙∙∙F and C_methyl_-H∙∙∙O interactions connect the complexes to a supramolecular network, in which the hydroxy group of the ethanol molecule participates in the formation of a 16-membered ring motif of the graph set R_4_^4^(16) [46,47,48,49].

The receptor cation of the hexafluorophosphate salt **3a** differs from the aforementioned case by the presence of an OH group in the *meta*-position of the pyridinium ring. This structural difference has a fundamental influence on the packing and coordination behavior of the molecules in the solid-state structure. The crystal growth of **3a** from methanol and water (1:1, *v*/*v*) yields colorless plates of the orthorhombic space group *Pna*2_1_ with two receptor cations, two PF_6_^−^ ions, one methanol molecule and three water molecules in the asymmetric unit of the cell [(**3^+^**PF_6_^−^)_2_**·**MeOH**·**(H_2_O)_3_; solvate **3S**]. These components are combined to form two different complexes **3S-I** (**3^+^**PF_6_^−^**·**H_2_O) and **3S-II** [**3^+^**PF_6_^−^**·**(H_2_O)_2_**·**CH_3_OH], the structures of which are shown in Figure 2c, Figure 3c and Figure 4c. In the case of complex **3S-I**, the geometry of **3^+^**PF_6_^−^ and the mode of cation–anion interaction resemble that of **1^+^**PF_6_^−^ and **2^+^**PF_6_^−^. The water molecule is connected by an O-H∙∙∙N bond [*d*(H∙∙∙N) 2.03 Å] to the pyrimidine-N-atom N4 of the receptor, while its second H atom acts as a trifurcated donor for O-H∙∙∙F bonding [*d*(H∙∙∙F) 2.28–2.51 Å] to the PF_6_^−^ ion.

In the second complex (**3S-II**), the cavity of the receptor cation is occupied by one methanol and two water molecules, which participate in the formation of a pattern of N-H∙∙∙O, O-H∙∙∙O and O-H∙∙∙N bonds with the amine H atoms and a pyrimidine N atom of the receptor [*d*(H∙∙∙N) 1.91 Å, *d*(H∙∙∙O) 1.86–2.49 Å]. The PF_6_^−^ ion is connected to a methyl H atom of the alcohol molecule by a weak C-H∙∙∙F bond [*d*(H∙∙∙F) 2.53 Å].

The two independent cations in the asymmetric unit of **3S** have slightly different conformations. These differences are expressed in Figure S1 (see Supporting Information), showing the superposition of the two molecules.

**Figure 7:**
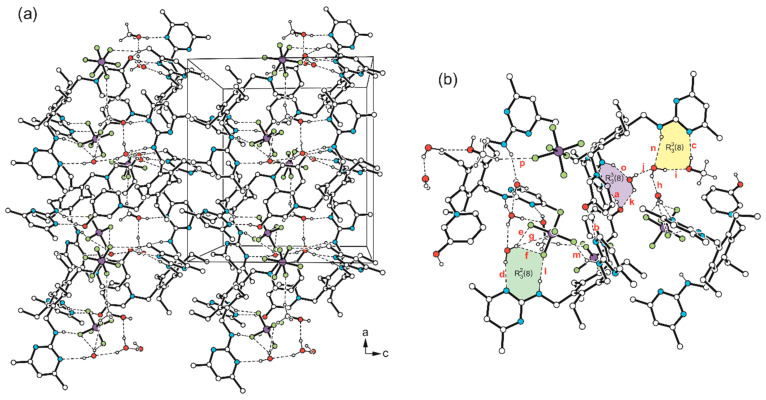
(**a**) Packing diagram of the complex **3a·**MeOH**·**H_2_O (2:1:3; **3S**) viewed down the crystallographic *b*-axis and (**b**) excerpt of the one-dimensional supramolecular aggregate. Dashed lines represent hydrogen bond interactions. In (**b**) the eight-membered ring motifs are marked by color highlighting. Nitrogen atoms are displayed as blue, oxygen as red, phosphorus as violet and fluorine atoms as green circles. In (**b**) the following interactions are shown: O-H∙∙∙N (a–d = 1.81–2.03 Å), O-H∙∙∙F (e–g = 2.28–2.51 Å), O-H∙∙∙O (h–k = 1.86–2.49 Å), N-H∙∙∙F (l, m = 2.41, 2.59 Å), N-H∙∙∙O (n, o = 2.18, 2.27 Å) and C-H∙∙∙F (p = 2.53 Å).

The solvent molecules, as well as the hydroxy group of the receptor, connect the complexes to one-dimensional structure domains that extend parallel to the crystallographic *a*-axis (Figure 7a). These aggregates are characterized by structurally different supramolecular ring motifs (see Figure 7b), which follow the graph sets R_3_^2^(8) and R_3_^3^(8).

#### 2.2.2. Hexafluorophosphate Salts of the Dicationic Compounds: Solvates **4S**, **5S** and **6S**

**Figure 8:**
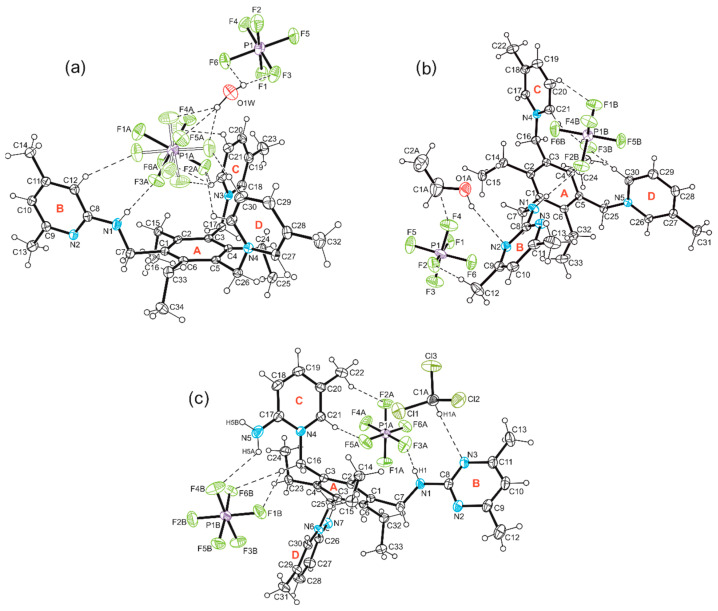
ORTEP diagrams of the molecular structures of the complexes **4a·**H_2_O (**4S**) (**a**), **5a·**EtOH (**5S**) (**b**) and **6a·**CHCl_3_ (**6S**) (**c**), including atom labeling and ring specification. Displacement ellipsoids are drawn at the 50% probability level. Dashed lines represent hydrogen bond interactions. For the sake of clarity, only the major disorder positions of the PF_6_^−^ ions in **5S** and **6S** are shown.

**Figure 9:**
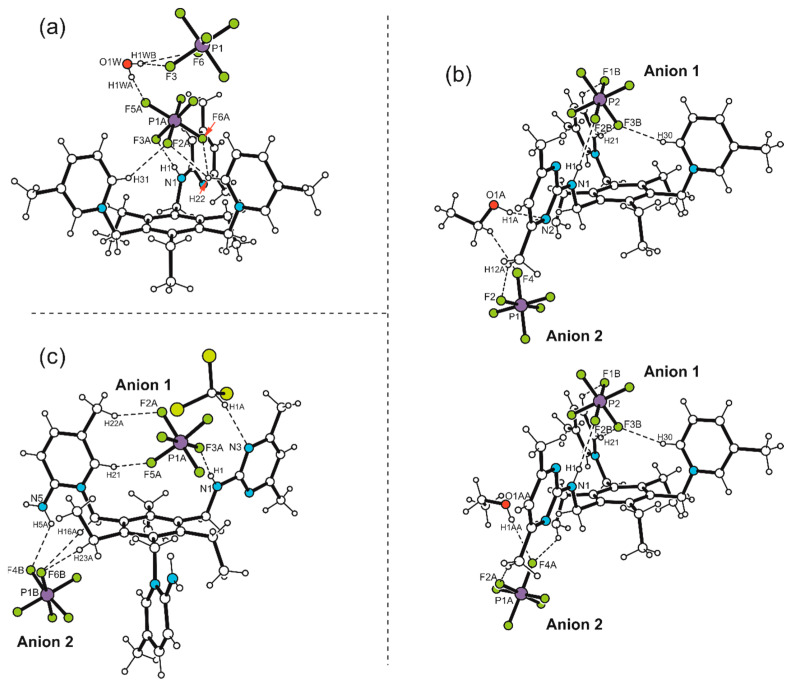
Side views (ball-and-stick models) of the complex structures **4a·**H_2_O (**4S**) (**a**), **5a·**EtOH (**5S**) (**b**) and **6a·**CHCl_3_ (**6S**) (**c**), including the labeling of coordinating atoms. In (**b**), the disorder components of the ethanol molecule and anion 2 are shown.

The crystals of **4a** obtained from acetonitrile and water (7:1, *v*/*v*) proved to be a mono-hydrate **4a·**H_2_O [solvate **4S**; space group *P*-1] with the asymmetric unit of the cell containing the dicationic receptor, two PF_6_^−^ ions and one molecule of water, which form a complex of the structure, shown in Figure 2d, Figure 8a and Figure 9a. The PF_6_^−^ ion accommodated in the cavity of the receptor is disordered over two positions (s.o.f. 0.85/0.15). The major disorder component of this anion interacts with the receptor by a N-H∙∙∙F [*d*(H∙∙∙F) 2.21(1) Å] and three C-H∙∙∙F bonds [*d*(H∙∙∙F) 2.39–2.55 Å], whereas the minor disorder position of this anion is involved in the formation of four C-H∙∙∙F bonds [*d*(H∙∙∙F) 2.33–2.54 Å]. The hydrogen atoms of the water molecule act as bifurcated binding sites for O-H∙∙∙F bonds [*d*(H∙∙∙F) 2.05(2)–2.54(3) Å] with the anions of the complex.

**Figure 10:**
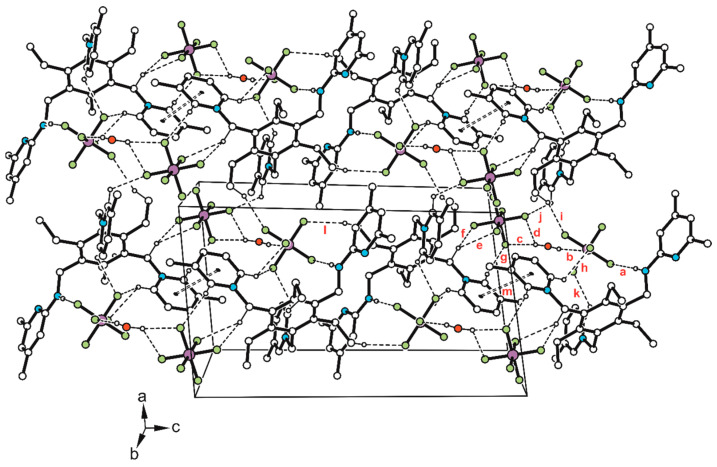
Packing diagram of the complex **4a·**H_2_O (**4S**). Dashed lines represent hydrogen bond interactions, dashed double lines represent *π*∙∙∙*π* stacking interactions. Nitrogen atoms are displayed as blue, oxygen as red, phosphorus as violet and fluorine atoms as green circles. The following interactions are shown: N-H∙∙∙F (a = 2.22 Å), O-H∙∙∙F (b–d = 2.05–2.54 Å), C-H∙∙∙F (e–l = 2.39–2.54 Å) and *π*∙∙∙*π* (m = 4.18 Å).

The complexes are linked by weak C_aryl_-H∙∙∙F type hydrogen bonds [*d*(H∙∙∙F) 2.31–2.58 Å] and offset *π*∙∙∙*π* (face-to-face) interactions [*d*(*Cg*∙∙∙*Cg*) 3.835(1)–4.186(1) Å and slippage 0.499–1.695 Å] to form a 3D network. An excerpt of the packing structure is presented in Figure 10.

Crystal growth of the salt **5a** from ethanol yields a solvate **5a·**EtOH (**5S**) of the space group *P*-1 with the receptor cation, two PF_6_^−^ ions, one of them disordered over two positions, and one twofold disordered ethanol molecule in the asymmetric part of the unit cell. The three functionalized side-arms of the receptor, two of which bear a 3-methylpyridinium group and one a dimethylpyrimidinylamino moiety, are arranged on the same face of the central benzene ring (*ab′ab′ab′* conformation). The aromatic rings of these units are inclined at angles of 51.8(1), 78.9(1) and 36.8(1)° to one another, indicating a less symmetric conformation. The PF_6_^−^ ion located inside the cavity of the receptor cation is connected by one N-H∙∙∙F [*d*(H∙∙∙F) 2.29(2) Å] and three C-H∙∙∙F hydrogen bonds [*d*(H∙∙∙F) 2.24–2.48 Å], the latter involving H atoms of the pyridinium groups. The second PF_6_^−^ ion is located outside the cavity.

**Figure 11:**
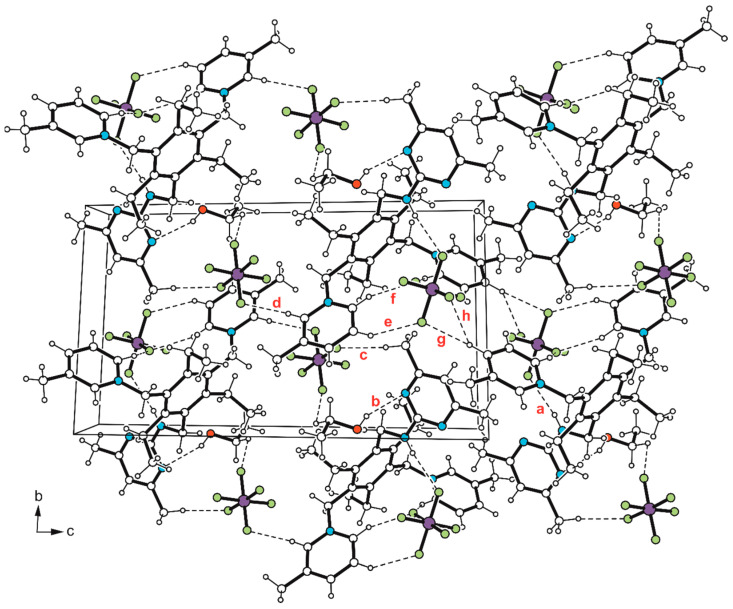
Packing diagram of the complex **5a·**EtOH (**5S**) viewed down the crystallographic *a*-axis. Dashed lines represent hydrogen bond interactions. Nitrogen atoms are displayed as blue, oxygen as red, phosphorus as violet and fluorine atoms as green circles. For the sake of clarity, only one position of the disordered anion/solvent is shown. The following interactions are shown: N-H···F (a = 2.29 Å), O-H···N (b = 2.49 Å) and C-H···F (c–h = 2.30–2.54 Å).

The two disorder positions of the alcohol molecule contribute in a different way to complex formation. In one case, its OH hydrogen atom is connected to the pyrimidine nitrogen N2 of the receptor [*d*(H∙∙∙N) 2.49 Å]. In the second disorder position of the solvent, the OH hydrogen is linked to the PF_6_^−^ ion (anion 2 in Figure 9b) via O-H∙∙∙F bonding [*d*(H···F) 2.36 Å]. A variety of C-H∙∙∙N [*d*(H∙∙∙N) 2.38–2.60 Å] and C-H∙∙∙F hydrogen bonds [*d*(H∙∙∙F) 2.43–2.55 Å] (see Figure 11) connect the complexes to a close three-dimensional network.

**Figure 12:**
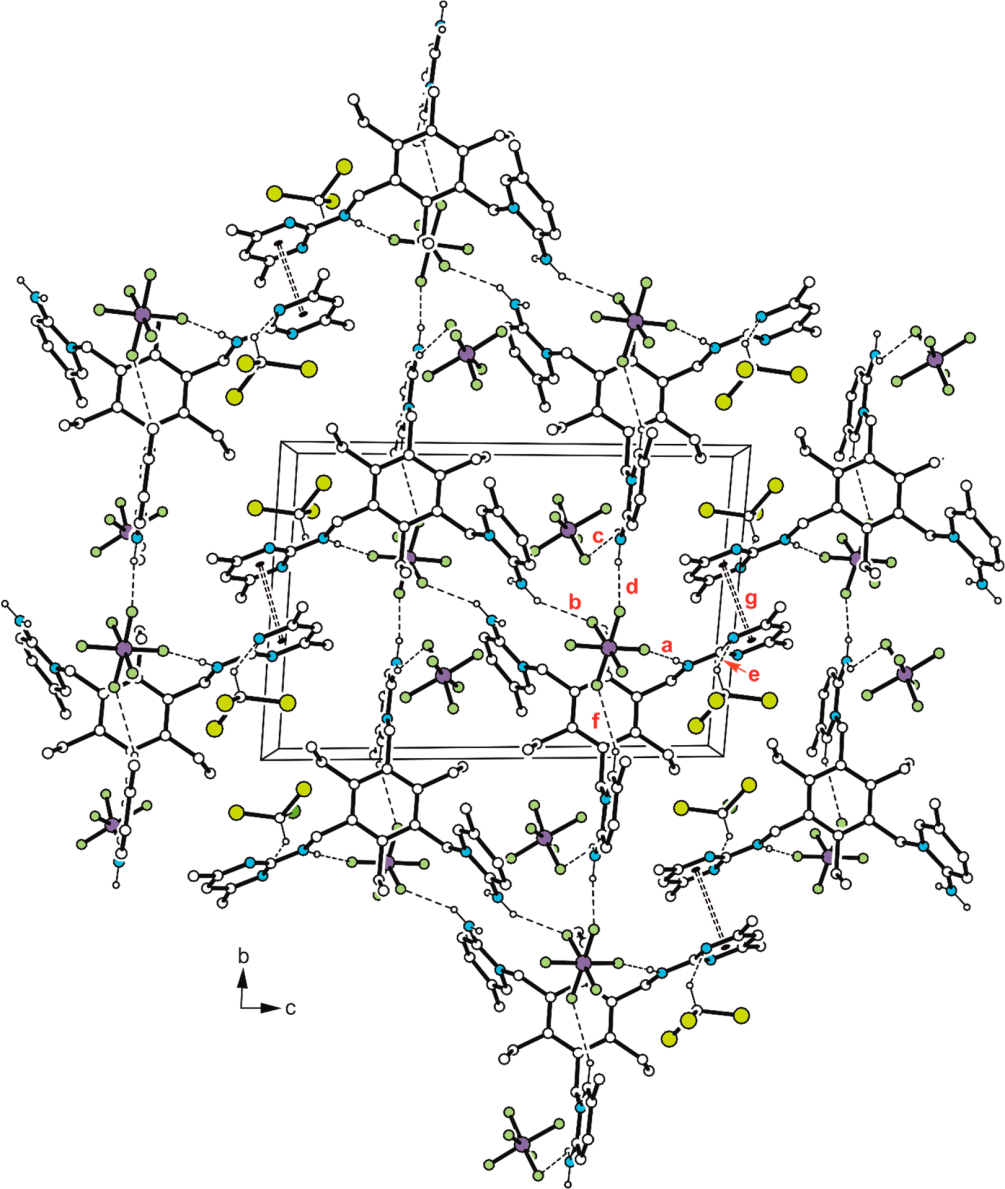
Packing diagram of the complex **6a·**CHCl_3_ (**6S**) viewed down the crystallographic *a*-axis. Nitrogen atoms are displayed as blue, phosphorus as violet, chlorine and fluorine atoms as green circles. Only one position of the disordered anions is illustrated. Dashed lines represent H bonds, dashed double lines represent *π*···*π* arene interactions. The following interactions are shown: N-H∙∙∙F (a = 2.26 Å, b = 2.06 Å, c = 2.66 Å, d = 2.22 Å), C-H∙∙∙N (e = 2.63 Å, f = 2.71 Å) and *π*∙∙∙*π* stacking (*Cg*∙∙∙*Cg* distance; g = 3.66 Å).

The colorless crystals of **6a** obtained from CHCl_3_ also proved to be a solvate **6a·**CHCl_3_ [solvate **6S**; space group *P*-1] with one molecule of the dicationic receptor, two disordered PF_6_^−^ counterions and one solvent molecule in the asymmetric unit of the cell [**6^2+^**(PF_6_^−^)_2_**·**CHCl_3_]. In the crystal, the receptor does not possess a preorganized binding pocket, as it displays a “*two-up, one-down*” arrangement of the functionalized arms. As is obvious from Figure 8c and Figure 9c, the two 2-amino-5-methylpyridinium fragments are located on opposite sides of the central arene ring with their amino groups pointing away from the central benzene ring. Taking into account the ethyl groups, the receptor adopts an *ab’ab’ba’* conformation. The PF_6_^−^ ions participate in different ways in complex formation. One of them (anion 1 in Figure 9c) is connected to the receptor by a N_amino_-H∙∙∙F bond [*d*(H···F) 2.26(2) Å] as well as C-H∙∙∙F interactions. The major disorder component of the second anion is linked to the amino hydrogen H5A [*d*(H∙∙∙F) 2.41 Å] and the bifurcated donor atom H16B [*d*(H∙∙∙F) 2.48, 2.54 Å]. The solvent molecule is associated by a C-H∙∙∙N bond with the pyrimidine-N atom N3 of the receptor [*d*(H∙∙∙N) 2.63 Å]. Viewing the crystal structure as in Figure 12 reveals that the complex molecules are connected to double strand-like supramolecular aggregates extending parallel to the crystallographic *b*-axis. Molecules of adjacent strands are connected by offset *π*∙∙∙*π* (face-to-face) interactions with a *Cg*∙∙∙*Cg* distance of 3.629(3) Å and a slippage of 0.858 Å between the interacting pyrimidine rings.

## 3. Conclusions

Crystal structures of the hexafluorophospate salts **1a**–**6a**, bearing pyridinium- and aminopyridine- or aminopyrimidine-based groups, show a tendency of these molecules to bind the PF_6_^−^ counterion in the cavity formed by the three functionalized side-arms (see Figure 2). The intermolecular interactions comprise N-H∙∙∙F and C-H∙∙∙F bonds, the latter involving mostly an *ortho*-H atom of the pyridinium group.

In the case of the monocationic compounds **1a**–**3a**, the only exception is represented by one of the two complexes observed in the crystal structure of the solvate **3S** (see Figure 2c), where the cavity of the receptor cation is occupied by one methanol and two water molecules [**3^+^**PF_6_^−^**·**(H_2_O)_2_**·**CH_3_OH, **3S-II**]. The presence of two complexes in the crystal structure **3S** [**3^+^**PF_6_^−^**·**H_2_O (**3S-I**) and **3^+^**PF_6_^−^**·**(H_2_O)_2_**·**CH_3_OH (**3S-II**)] represents an interesting finding. It is worth noting that the presence of two types of complexes was also observed by us in the crystal structures formed by acyclic receptors and glucopyranosides [50,51], as well as for ammonium receptors [52].

Among the dicationic compounds **4a**–**6a**, the molecular cation **6**^2**+**^ does not possess a preorganized binding pocket (Figure 2f), because the two pyridinium groups project in opposite directions with respect to the central benzene ring (the molecule adopts an *ab′ab′ba′* conformation; *a* = above, *b* = below, *a′*/*b′* = ethyl). In all other cases (**1a**, **2S**, **3S-I**, **3S-II**, **4S** and **5S**) the arrangement of the substituents around the benzene ring follows an *ab′ab′ab′* pattern.

The replacement of the pyridinyl (compound **1a** and **4a**) by pyrimidinyl group(s) (compound **2a** and **5a**, respectively) does not cause any significant changes in terms of the conformation and interactions of the receptor cation with the counterion (see **1a** vs. **2S** and **4S** vs. **5S**).

The obtained results suggest that, for receptor molecules of the type studied, other counterions, e.g., tetraphenylborate, should also be considered in molecular recognition studies of anionic substrates.

The detailed analysis of the various supramolecular motifs observed in the crystal structures of the hexafluorophosphate salts **1a**–**6a**, including those involving solvent molecules, provides valuable information on the interactions of the receptor cation with the counterion, gives deeper insights into the processes of molecular recognition, and is helpful in the development of new receptor molecules for anions and in the selection of the most suitable counterion.

## 4. Experimental Section

Commercially available starting materials were purchased from Sigma-Aldrich (2-amino-4,6-dimethylpyrimidine, 3-hydroxypyridine, 2-amino-5-methylpyridine), TCI (2-amino-4,6-dimethylpyridine) and Acros Organics (3-methylpyridine). Melting points were measured on a hot stage microscope (Büchi 510) and are uncorrected. ^1^H and ^13^C NMR spectra were recorded on a Bruker Avance III-500 MHz or Jeol Resonance ECZ500R NMR spectrometer using Me_4_Si as an internal standard. Mass spectra were recorded on a Bruker amazon SL in combination with a Thermo Ultimate 3000 HPLC System (column: Thermo Acclaim_TM_ 120, eluent: CH_3_OH/H_2_O + 0.1% HCOOH). The elemental analysis was performed on a vario MICRO cube (Elementar Analysesysteme GmbH).

Synthesis of compounds **1b**–**6b** and **1a**–**6a**, **7**–**10**. For a general overview of the synthesis routes described below, see Scheme 1. Precursor compounds **7**–**10** were prepared from 1,3,5-tris(bromomethyl)-2,4,6-triethylbenzene and 2-amino-4,6-dimethylpyrimidine or 2-amino-4,6-dimethylpyridine according to the procedure reported by us previously [35,36]. Compounds **1a**, **1b**, **4a** and **4b** were prepared according to modified synthesis procedures with respect to ref. [9].

*1-[(3-methylpyridinium-1-yl)methyl]-3,5-bis[(4,6-dimethylpyridin-2-yl)aminomethyl]-2,4,6-triethylbenzene bromide* (**1b**). 3-Methylpyridine (0.056 mL, 0.571 mmol) was added to a solution of **7** (250 mg, 0.48 mmol) in chloroform (20 mL) and the reaction mixture was refluxed for 2 d. The solvent was evaporated, and the crude product purified by column chromatography (chloroform/methanol (4:1, *v*/*v*)). Yield: 40% (130 mg, 0.19 mmol). ^1^H NMR (500 MHz, CD_3_OD): *δ =* 1.13 (t, *J* = 7.5 Hz, 6H), 1.29 (t, *J* = 7.4 Hz, 3H), 2.26 (s, 6H), 2.40 (s, 6H), 2.62 (s, 3H), 2.72 (q, *J* = 7.4 Hz, 4H), 2.86 (q, *J* = 7.5 Hz, 2H), 4.53 (s, 4H), 5.99 (s, 2H), 6.46 (s, 2H), 6.49 (s, 2H), 8.00 (dd, *J* = 8.0 Hz/6.2 Hz, 1H), 8.45 (d, *J* = 7.9 Hz, 1H), 8.59 (d, *J* = 6.1 Hz, 1H), 8.87 (s, 1H) ppm. ^13^C NMR [125 MHz, CD_3_OD/DMSO-*d*_6_ (5:2, *v*/*v*)]: *δ =* 16.5, 16.8, 18.6, 21.1, 23.8, 24.2, 24.4, 41.4, 59.4, 106.3, 114.7, 126.3, 129.0, 135.8, 141.5, 142.0, 144.9, 146.6, 147.8, 148.8, 150.6, 157.0, 160.0 ppm.

*1-[(3-methylpyridinium-1-yl)methyl]-3,5-bis[(4,6-dimethylpyridin-2-yl)aminomethyl]-2,4,6-triethylbenzene hexafluorophosphate* (**1a**). A solution of NaPF_6_ (120 mg, 0.71 mmol) was added to a solution of **1b** (145 mg, 0.24 mmol) in water and methanol (10 mL (2:3, *v*/*v*)) and stirred for 2 d at room temperature, whereby the pure product precipitated as a white solid which was filtered off and washed with water and diethyl ether. Yield: 62% (100 mg, 0.15 mmol). M.p. 219–221 °C. ^1^H NMR [500 MHz, CD_3_OD/CD_3_CN (6:0.1, (*v*/*v*)]: *δ =* 1.11 (t, *J* = 7.4 Hz, 6H), 1.28 (t, *J* = 7.5 Hz, 3H), 2.22 (s, 6H), 2.34 (s, 6H), 2.59 (s, 3H), 2.70 (q, *J* = 7.4 Hz, 4H), 2.86 (q, *J* = 7.5 Hz, 2H), 4.48 (s, 4H), 5.92 (s, 2H), 6.30 (s, 2H), 6.39 (s, 2H), 7.95 (dd, *J* = 8.0 Hz/6.1 Hz, 1H), 8.40–8.45 (m, 2H), 8.71 (s, 1H) ppm. ^13^C NMR [125 MHz, CD_3_OD/CD_3_CN (6:0.1, *v*/*v*)]: *δ =* 16.4, 16.7, 18.5, 21.1, 23.5, 24.1, 24.3, 41.4, 59.3, 106.2, 114.7, 126.1, 128.9, 135.6, 141.5, 141.7, 144.7, 146.6, 147.8, 148.8, 151.1, 156.8, 159.5 ppm. LCMS (ESI) *m*/*z* calcd. for C_35_H_46_N_5_PF_6_: 536.37 [M^+^], 682.35 [M^+^ + H^+^ + PF_6_^−^], found 536.41, 682.42. Anal. calcd. (%) for C_35_H_46_N_5_PF_6_·H_2_O: C 60.07, H 6.91, N 10.01, found: C 60.05, H 6.64, N 10.03.

*1-[(3-methylpyridinium-1-yl)methyl]-3,5-bis[(4,6-dimethylpyrimidin-2-yl)aminomethyl]-2,4,6-triethylbenzene bromide* (**2b**). Precursor **8** (200 mg, 0.38 mmol) was dissolved in tetrahydrofuran (10 mL) and 3-methylpyridine (0.044 mL, 0.45 mmol) was added dropwise. The resulting mixture was refluxed for 8 h and then stirred at room temperature for an additional 4 h. The precipitated white product was filtered off, washed with tetrahydrofuran and dried in a vacuum at 60 °C. Yield: 70% (165 mg, 0.27 mmol). M.p. 210–212 °C. ^1^H NMR (500 MHz, CD_3_OD): *δ =* 1.10 (t, *J* = 7.5 Hz, 6H), 1.27 (t, *J* = 7.4 Hz, 3H), 2.31 (s, 12H), 2.60 (s, 3H), 2.74 (q, *J* = 7.5 Hz, 4H), 2.89 (q, *J* = 7.5 Hz, 2H), 4.64 (s, 4H), 5.99 (s, 2H), 6.50 (s, 2H), 7.97 (dd, *J* = 8.0 Hz/6.1 Hz, 1H), 8.45 (d, *J* = 8.0 Hz, 1H), 8.54 (d, *J* = 6.2 Hz, 1H), 8.81 (s, 1H) ppm. ^13^C NMR [125 MHz, CD_3_OD/CDCl_3_ (5:2, *v*/*v*)]: *δ =* 14.8, 15.1, 17.0, 22.2, 22.5, 22.7, 39.0, 57.6, 109.3, 124.2, 127.2, 133.6, 139.7, 140.0, 142.8, 144.9, 146.0, 147.2, 160.8, 167.4 ppm. HRMS (ESI) *m*/*z* calcd. for C_33_H_44_N_7_Br: 538.3653 [M^+^], found 538.3655. Anal. calcd. (%) for C_33_H_44_N_7_Br·H_2_O: C 62.25, H 7.28, N 15.40, found: C 62.11, H 7.38, N 15.47.

*1-[(3-methylpyridinium-1-yl)methyl]-3,5-bis[(4,6-dimethylpyrimidin-2-yl)aminomethyl]-2,4,6-triethylbenzene hexafluorophosphate* (**2a**). A solution of NaPF_6_ (100 mg, 0.60 mmol) in methanol (4 mL) was added to a solution of **2b** (120 mg, 0.19 mmol) in a mixture of ethanol (10 mL) and methanol (1 mL) and stirred for 24 h at room temperature. The solvent was evaporated, and the crude product was purified by recrystallization from ethanol, yielding the product as a white solid. Yield: 53% (70 mg, 0.10 mmol). M.p. 235–238 °C. ^1^H NMR (500 MHz, DMSO-*d*_6_): *δ* = 0.99 (t, *J* = 7.4 Hz, 6H), 1.16 (t, *J* = 7.4 Hz, 3H), 2.20 (s, 12H), 2.53 (s, 3H), 2.62 (q, *J* = 7.6 Hz, 4H), 2.68 (q, *J* = 7.4 Hz, 2H), 4.46 (d, *J* = 4.4 Hz, 4H), 5.85 (s, 2H), 6.39 (s, 2H), 6.87 (t, *J* = 4.5 Hz, 2H), 7.99 (dd, *J* = 8.0 Hz/6.1 Hz, 1H), 8.46 (d, *J* = 7.9 Hz, 1H), 8.52 (d, *J* = 6.2 Hz, 1H), 8.85 (s, 1H) ppm. ^13^C NMR (125 MHz, DMSO-*d*_6_): *δ =* 16.05, 16.10, 18.0, 22.7, 23.1, 23.5, 39.1, 57.5, 108.9, 124.7, 127.4, 133.7, 138.7, 141.0, 143.7, 144.7, 146.1, 146.2, 161.7, 166.8 ppm. HRMS (ESI) *m*/*z* calcd. for C_33_H_44_N_7_PF_6_: 269.6863 [M^+^ + H^+^], 538.3653 [M^+^], 684.3373 [M^+^ + H^+^ + PF_6_^−^], found 269.6844, 538.3649, 684.3377. Anal. calcd. (%) for C_33_H_44_N_7_PF_6_: C 57.97, H 6.49, N 14.34, found: C 57.74, H 6.62, N 14.27.

*1-(3-hydroxypyridinium-1-yl)-3,5-bis[(4,6-dimethylpyrimidin-2-yl)aminomethyl]-2,4,6-triethylbenzene bromide* (**3b**). A solution of 3-hydroxypyridine (30 mg, 0.31 mmol) in toluene and methanol (4 mL (1:1, *v*/*v*)) was added to a hot solution of **8** (150 mg, 0.29 mmol) in toluene (4 mL) and refluxed for 8 h. After the reaction had finished, the white product was separated by filtration and washed with toluene and diethyl ether. Yield: 92% (163 mg, 0.26 mmol). M.p. 218–220 °C. ^1^H NMR (500 MHz, CD_3_OD): *δ* = 1.10 (t, *J* = 7.5 Hz, 6H), 1.26 (t, *J* = 7.5 Hz, 3H), 2.31 (s, 12H), 2.75 (q, *J* = 7.5 Hz, 4H), 2.90 (q, *J* = 7.5 Hz, 2H), 4.65 (s, 4H), 5.93 (s, 2H), 6.51 (s, 2H), 7.85 (dd, *J* = 8.7 Hz/5.6 Hz, 1H), 7.89 (ddd, *J* = 8.8 Hz/2.5 Hz/1.2 Hz, 1H), 8.22 (dt, *J* = 5.7 Hz/1.3 Hz, 1H), 8.23 (d, *J* = 2.3 Hz, 1H) ppm. ^13^C NMR (125 MHz, CD_3_OD): *δ* = 16.3, 16.7, 23.6, 24.25, 24.34, 40.8, 59.3, 110.9, 126.6, 129.8, 133.15, 133.19, 135.0, 135.4, 146.9, 148.9, 160.5, 162.6, 169.4 ppm. HRMS (ESI) *m*/*z* calcd. for C_32_H_42_N_7_OBr: 540.3445 [M^+^], found 540.3451. Anal. calcd. (%) for C_32_H_42_N_7_OBr·H_2_O: C 60.18, H 6.94, N 15.35, found: C 59.86, H 6.76, N 15.19.

*1-(3-hydroxypyridinium-1-yl)-3,5-bis[(4,6-dimethylpyrimidin-2-yl)aminomethyl]-2,4,6-triethylbenzene hexafluorophosphate* (**3a**). A suspension of **3b** (298 mg, 0.48 mmol) in water (4 mL) was heated up to 50 °C and mixed with methanol until it became a clear solution. After adding NaPF_6_ (251 mg, 1.49 mmol), dissolved in 0.6 mL water, the reaction mixture was stirred for 2 h at 50 °C followed by additional stirring at room temperature overnight. The solution was reduced to half the volume, whereby the product precipitated as a white solid which was filtered and washed with water and diethyl ether. Yield: 82% (270 mg, 0.39 mmol). M.p. 174–176 °C. ^1^H NMR (500 MHz, CD_3_OD): *δ* = 1.10 (t, *J* = 7.5 Hz, 6H), 1.26 (t, *J* = 7.5 Hz, 3H), 2.31 (s, 12H), 2.75 (q, *J* = 7.5 Hz, 4H), 2.89 (q, *J* = 7.5 Hz, 2H), 4.64 (s, 4H), 5.87 (s, 2H), 6.49 (s, 2H), 7.72–7.79 (m, 2H), 8.04–8.10 (m, 2H) ppm. ^13^C NMR (125 MHz, CD_3_OD): *δ* = 16.3, 16.7, 23.7, 24.25, 24.32, 40.8, 59.1, 110.8, 126.8, 129.5, 133.2, 133.3, 133.4, 135.4, 146.8, 148.8, 162.5, 162.8, 169.4 ppm. HRMS (ESI) *m*/*z* calcd. for C_32_H_42_N_7_OPF_6_: 540.3445 [M^+^], found 540.3454. Anal. calcd. (%) for C_32_H_42_N_7_OPF_6_·H_2_O: C 54.62, H 6.30, N 13.93, found: C 54.64, H 6.34, N 14.07.

*1,3-bis[(3-methylpyridinium-1-yl)methyl]-5-[(4,6-dimethylpyridin-2-yl)aminomethyl]-2,4,6-triethylbenzene bromide* (**4b**). After the addition of 3-methylpyridine (0.13 mL, 1.33 mmol) to a solution of **9** (290 mg, 0.60 mmol) in acetone (15 mL), the reaction mixture was refluxed for 2 h before it was allowed to slowly cool down to room temperature. During that process, a white precipitate formed which was filtered off and washed with acetone. In case of remaining impurities, the solid can be taken up in small amounts of methanol and precipitated with THF again. Yield: 60% (240 mg, 0.36 mmol). ^1^H NMR (500 MHz, CD_3_OD): *δ* = 1.03 (t, *J* = 7.5 Hz, 3H), 1.25 (t, *J* = 7.5 Hz, 6H), 2.38 (s, 3H), 2.54 (q, *J* = 7.5 Hz, 2H), 2.55 (s, 3H), 2.64 (s, 6H), 2.75 (q, *J* = 7.5 Hz, 4H), 4.56 (s, 2H), 6.03 (s, 4H), 6.67 (s, 1H), 7.05 (s, 1H), 8.00 (dd, *J* = 8.0 Hz/6.2 Hz, 2H), 8.43 (d, *J* = 8.0 Hz, 2H), 8.81 (d, *J* = 6.2 Hz, 2H), 8.93 (s, 2H) ppm. ^13^C NMR (125 MHz, CD_3_OD): *δ* = 15.8, 16.3, 18.8, 19.1, 21.9, 24.79, 24.84, 42.0, 58.8, 110.4, 115.9, 127.9, 129.2, 133.4, 141.6, 142.5, 144.7, 147.7, 147.9, 149.4, 151.0, 154.1 ppm.

*1,3-bis[(3-methylpyridinium-1-yl)methyl]-5-[(4,6-dimethylpyridin-2-yl)aminomethyl]-2,4,6-triethylbenzene hexafluorophosphate* (**4a**). To a solution of **4b** (220 mg, 0.19 mmol) in methanol (6 mL), a solution of NaPF_6_ in methanol (3 mL) was added. The resulting mixture was stirred at room temperature for 2 d, the precipitated white solid was filtered, washed with water and diethyl ether, and carefully dried under high vacuum. Yield: 92% (140 mg, 0.78 mmol). M.p. 173–176 °C. ^1^H NMR [500 MHz, CD_3_OD/CD_3_CN (2:1, *v*/*v*)]: *δ* = 0.97 (t, *J* = 7.5 Hz, 3H), 1.17 (t, *J* = 7.5 Hz, 6H), 2.31 (s, 3H), 2.46 (s, 3H), 2.58 (q, *J* = 7.6 Hz, 2H), 2.59 (s, 6H), 2.75 (q, *J* = 7.5 Hz, 4H), 4.36 (s, 2H), 5.94 (s, 4H), 6.57 (s, 1H), 6.60 (s, 1H), 7.95 (dd, *J* = 8.0 Hz/6.1 Hz, 2H), 8.36 (d, *J* = 6.0 Hz, 2H), 8.42 (d, *J* = 7.9 Hz, 2H), 8.75 (s, 2H) ppm. ^13^C NMR [125 MHz, CD_3_OD/CD_3_CN (2:1, (*v*/*v*)]: *δ* = 15.5, 16.0, 18.5, 20.8, 21.6, 24.6, 24.7, 41.5, 58.9, 109.0, 115.5, 127.9, 129.1, 135.0, 141.5, 141.6, 144.9, 147.9, 149.1, 150.7, 151.2, 155.4, 156.0 ppm. LCMS (ESI) *m*/*z* calcd. For C_34_H_44_N_4_P_2_F_12_: 254.18 [M^2+^], 653.32 [M^2+^ + PF_6_^−^]^+^ found 254.41, 653.49. Anal. Calcd. (%) for C_34_H_44_N_4_P_2_F_12_·H^+^·PF_6_^−^·H_2_O: C 42.50, H 4.92, N 5.82 found: C 42.50, H 5.06, N 5.83.

*1,3-bis[(3-methylpyridinium-1-yl)methyl]-5-[(4,6-dimethylpyrimidin-2-yl)aminomethyl]-2,4,6-triethylbenzene bromide* (**5b**). Precursor **10** (200 mg, 0.42 mmol) was dissolved in dichloromethane (13 mL), mixed with 3-methylpyridine (0.095 mL, 0.97 mmol), and stirred for 8 h at boiling temperature and another 4 h at room temperature to complete the precipitation process. The white solid was filtered and taken up again in dichloromethane to wash out all impurities. Yield: 86% (248 mg, 0.36 mmol). M.p. 230–232 °C. ^1^H NMR (500 MHz, CD_3_OD): *δ* = 0.88 (t, *J* = 7.5 Hz, 3H), 1.14 (t, *J* = 7.5 Hz, 6H), 2.30 (s, 6H), 2.62 (s, 6H), 2.69 (q, *J* = 7.6 Hz, 2H), 2.83 (q, *J* = 7.5 Hz, 4H), 4.69 (s, 2H), 6.04 (s, 4H), 6.49 (s, 1H), 8.00 (dd, *J* = 8.0 Hz/6.1 Hz, 2H), 8.46 (d, *J* = 8.0 Hz, 2H), 8.61 (d, *J* = 6.2 Hz, 2H), 8.91 (s, 2H) ppm. ^13^C NMR (125 MHz, CD_3_OD): *δ* = 15.8, 16.1, 18.6, 23.7, 24.8, 25.0, 40.8, 59.2, 110.9, 128.0, 129.2, 137.0, 141.7, 142.0, 145.0, 147.9, 148.4, 150.6, 163.0, 169.3 ppm. HRMS (ESI) *m*/*z* calcd. For C_33_H_43_N_5_Br_2_: 254.6754 [M^2+^], 590.2681 [M^+^ + Br^−^] found 254.6745, 590.2707. Anal. Calcd. (%) for C_33_H_43_N_5_Br_2_·H_2_O: C 57.65, H 6.60, N 10.19, found: C 57.79, H 6.97, N 10.15.

*1,3-bis[(3-methylpyridinium-1-yl)methyl]-5-[(4,6-dimethylpyrimidin-2-yl)aminomethyl]-2,4,6-triethylbenzene hexafluorophosphate* (**5a**). To a solution of **5b** (127 mg, 0.19 mmol) in methanol (5 mL), a solution of NaPF_6_ (199 mg, 1.19 mmol) in methanol (3 mL) was added dropwise. After stirring at room temperature for 24 h, the white precipitate was filtered off, taken up in methanol for further washing, filtered again, and dried in vacuo at 60 °C. Yield: 63% (95 mg, 0.12 mmol). M.p. 160–162 °C. ^1^H NMR [500 MHz, CD_3_OD/DMSO-*d*_6_ (5:2, *v*/*v*)]: *δ* = 0.92 (t, *J* = 7.4 Hz, 3H), 1.15 (t, *J* = 7.5 Hz, 6H), 2.52 (s, 6H), 2.64 (s, 6H), 2.68 (q, *J* = 7.5 Hz, 2H), 2.85 (q, *J* = 7.5 Hz, 4H), 4.81 (s, 2H), 6.04 (s, 4H), 6.85 (s, 1H), 8.03 (dd, *J* = 8.0 Hz/6.2 Hz, 2H), 8.49 (d, *J* = 6.1 Hz, 2H), 8.51 (d, *J* = 7.9 Hz, 2H), 8.87 (s, 1H) ppm. ^13^C NMR [125 MHz, CD_3_OD/DMSO-*d*_6_ (5:2, *v*/*v*)]: *δ* = 15.9, 16.2, 18.8, 22.9, 24.7, 24.8, 40.9, 58.9, 111.7, 128.2, 129.1, 134.6, 141.4, 141.6, 145.0, 148.0, 149.0, 150.6, 161.3, 168.2 ppm. HRMS (ESI) *m*/*z* calcd. For C_33_H_43_N_5_P_2_F_12_: 254.6754 [M^2+^], found 254.6744. Anal. Calcd. (%) for C_33_H_43_N_5_P_2_F_12_·H^+^·PF_6_^−^: C 41.13, H 4.81, N 7.27 found: C 41.29, H 4.67, N 7.22.

*1,3-bis[(2-amino-5-methylpyridinium-1-yl)methyl]-5-[(4,6-dimethylpyrimidin-2-yl)aminomethyl]-2,4,6-triethylbenzene bromide* (**6b**). A solution of 2-amino-5-methylpyridine (103 mg, 0.94 mmol) in dichloromethane (3 mL) was added dropwise to a solution of **10** (200 mg, 0.42 mmol) in dichloromethane (8 mL). The reaction mixture was refluxed for 15 h and then stirred for an additional 4 h at room temperature. The product precipitated as a white solid, was filtered and washed with dichloromethane to give pure 6b. Yield: 62% (180 mg, 0.26 mmol). M.p. 200–202 °C. ^1^H NMR (500 MHz, CD_3_OD): *δ* = 1.09 (bt, *J* = 7.1 Hz, 3H), 1.26 (t, *J* = 7.5 Hz, 6H), 2.14 (s, 6H), 2.32 (s, 6H), 2.42 (bq, *J* = 7.2 Hz, 2H), 2.76 (s, 4H), 4.69 (s, 2H), 5.26 (s, 4H), 6.51 (s, 1H), 6.98 (d, *J* = 2.0 Hz, 2H), 7.17 (d, *J* = 9.0 Hz, 2H), 7.79 (dd, *J* = 9.1 Hz/2.0 Hz, 2H) ppm. ^13^C NMR (125 MHz, CD_3_OD): *δ* = 15.4, 16.1, 17.4, 23.7, 24.4, 24.6, 40.8, 50.8, 110.9, 116.3, 125.3, 127.7, 133.2, 137.2, 145.6, 147.5, 150.1, 155.2, 162.9, 169.4 ppm. HRMS (ESI) *m*/*z* calcd. for C_33_H_45_N_7_Br_2_: 269.6863 [M^2+^], found 269.6843. Anal. calcd. (%) for C_33_H_45_N_7_Br_2_: C 56.66, H 6.48, N 14.02, found: C 56.30, H 6.49, N 13.98. (Abbreviations:, bt–broad triplet, bq–broad quartet).

*1,3-bis[(2-amino-5-methylpyridinium-1-yl)methyl]-5-[(4,6-dimethylpyrimidin-2-yl)aminomethyl]-2,4,6-triethylbenzene hexafluorophosphate* (**6a**). Bromide salt **6b** (110 mg, 0.16 mmol) was suspended in water (10 mL) and stirred thoroughly at 35 °C until completely dissolved. An aqueous solution of NaPF_6_ (166 mg, 0.99 mmol, 2 mL water) was added, and **6a** immediately precipitated as a white solid. After stirring at 45 °C for 3 h and additional stirring at room temperature overnight, the solid was filtered, washed with water and diethyl ether, and dried in vacuo at 60 °C for 2 d. Yield: 63% (97 mg, 0.12 mmol). M.p. 223–224 °C. ^1^H NMR (500 MHz, CD_3_OD): *δ* = 1.09 (bt, *J* = 7.1 Hz, 3H), 1.26 (t, *J* = 7.5 Hz, 6H), 2.13 (s, 6H), 2.34 (s, 6H), 2.40 (q, *J* = 7.2 Hz, 2H), 2.76 (s, 4H), 4.71 (s, 2H), 5.23 (s, 4H), 6.56 (s, 1H), 6.94–7.02 (m, 2H), 7.17 (d, *J* = 9.0 Hz, 2H), 7.79 (dd, *J* = 9.1 Hz/2.0 Hz, 2H) ppm. ^13^C NMR (125 MHz, CD_3_OD): *δ* = 15.3, 16.0, 17.3, 23.5, 24.3, 24.6, 40.8, 50.6, 111.1, 116.3, 125.4, 127.8, 133.2, 136.8, 145.7, 147.7, 150.1, 155.1, 162.2, 169.3 ppm. HRMS (ESI) *m*/*z* calcd. for C_33_H_45_N_7_P_2_F_12_: 269.6863 [M^2+^], 684.3373 [M^2+^ + PF_6_^−^], found 269.6846, 684.3382.

### X-ray Crystallography

The intensity data of **2S** was collected on a Smart APEX II diffractometer (Bruker AXS) with MoK_α_ radiation (λ = 0.71073 Å) using *ω-* and *ϕ*-scans. Data integration and reduction were processed with SAINT-NT [53]. An empirical absorption correction was applied to the collected reflections with SADABS [53]. Preliminary structure models were derived by the application of direct methods [54] and were refined by a full-matrix least-squares calculation based on *F*^2^ for all reflections. The intensity data of **1a**, **3S**, **4S**, **5S** and **6S** were collected on a STOE IPDS 2T diffractometer with MoK_α_ radiation (λ = 0.71073 Å) using the rotation method. The data reduction was processed with X-RED [55]. An empirical absorption correction was applied to the collected reflections with STOE X-SHAPE [55]. Preliminary structure models were derived by the application of direct methods using SIR2014 [56]. The non-hydrogen atoms of the cationic receptors were refined with anisotropic thermal parameters. The atoms of the highly disordered anions in **1a**, **2S** and **3S** were refined isotropically. With the exception of amino hydrogens, the OH hydrogens of the alcohol and water molecules in **2S**, **3S**, **4S** and **5S**, all other hydrogen atoms were included in the models in calculated positions and were refined as constrained to bonding atoms.

Crystallographic data for the structures described in this paper were deposited at the Cambridge Crystallographic Data Centre (CCDC) under identification numbers 2261366 (**1a**), 2261367 (**2S**), 2261368 (**3S**), 2261369 (**4S**), 2261370 (**5S**) and 2261371 (**6S**).

## Data Availability

The data presented in this study are available on request from the corresponding author.

## Supplementary Materials

The following supporting information can be downloaded at: https://www.mdpi.com/article/10.3390/molecules28186485/s1, Table S1: Crystallographic and structure refinement data of the crystal structures of **1a**, **2S** (**2a·**EtOH), **3S** [**3a·**MeOH**·**H_2_O (2:1:3)], **4S** (**4a·**H_2_O), **5S** (**5a·**EtOH) and **6S** (**6a·**CHCl_3_). Table S2: Relevant conformational parameters of the crystal structures of **1a**, **2S** (**2a·**EtOH), **3S** [**3a·**MeOH**·**H_2_O (2:1:3)], **4S** (**4a·**H_2_O), **5S** (**5a·**EtOH) and **6S** (**6a·**CHCl_3_). Table S3: Geometric parameters for non-covalent interactions in the crystal structures examined. Figure S1: Superposition of the two receptor cations found in the crystal structure **3S**. Figures S2a–S13a and S2b–S13b: ^1^H and ^13^C NMR spectra of compounds **1a**–**6a** and **1b**–**6b**.

## Sample Availability

Samples of all obtained compounds are available from the authors.
