# Supplementary material for: Supramolecular Motifs in the Crystal Structures of Triethylbenzene Derivatives Bearing Pyridinium Subunits in Combination with Pyrimidinyl or Pyridinyl Groups
Source: Molecules. 2023 Sep 7;28(18):6485. doi: 10.3390/molecules28186485 (PMC10535844; doi:10.3390/molecules28186485)
Supplement: Supplementary file 1 [file molecules-28-06485-s001.zip › molecules-2523534-supplementary.pdf]

Supporting Information

Table S1: Crystallographic and structure refinement data of the crystal structures of **1a**, **2S** (**2a**•EtOH), **3S** [**3a**•MeOH•H_2O (2:1:3)], **4S** (**4a**•H_2O), **5S** (**5a**•EtOH) and **6S** (**6a**•$CHCl_3$).

Table S2: Relevant conformational parameters of the crystal structures of **1a**, **2S** (**2a**•EtOH), **3S** [**3a**•MeOH•H_2O (2:1:3)], **4S** (**4a**•H_2O), **5S** (**5a**•EtOH) and **6S** (**6a**•$CHCl_3$).

Table S3: Geometric parameters for noncovalent interactions in the crystal structures examined.

Figure S1: Superposition of the two receptor cations found in the crystal structure **3S**.

Figures S2a–S13a and **S2b–S13b:** ^{1}H and ^{13}C NMR spectra of compounds **1a–6a** and **1b–6b**.

Table S1. Crystallographic and structure refinement data of the crystal structures of **1a**, **2S** (**2a**•EtOH), **3S** [**3a**•MeOH•H_2O (2:1:3)], **4S** (**4a**•H_2O), **5S** (**5a**•EtOH) and **6S** (**6a**•$CHCl_3$).

Compound	**1a**	**2S**	**3S**
Empirical formula	$C_{35}H_{46}N_5 \cdot PF_6$	$C_{33}H_{44}N_7 \cdot PF_6 \cdot C_2H_6O$	2 $C_{32}H_{42}N_6O \cdot 2\ PF_6 \cdot CH_4O \cdot 3\ H_2O$
Formula weight	681.74	729.79	1457.48
Crystal system	Triclinic	Triclinic	Orthorhombic
Space group	*P*-1	*P*-1	$Pna2_1$
a (Å)	9.0848(6)	9.1797(2)	16.5620(3)
b (Å)	11.3576(8)	12.1245(3)	29.1091(7)
c (Å)	18.7712(12)	18.4503(5)	15.0543(3)
α(°)	105.948(5)	97.596(1)	90.0
β(°)	92.268(5)	102.014(1)	90.0
γ (°)	105.808(5)	111.574(1)	90.0
V ($Å^3$)	1778.1(2)	1817.97(8)	7257.8(3)
Z	2	2	4
F(000)	720	772	3072
D_c (Mg m^{-3})	1.273	1.333	1.334
μ (mm^{-1})	0.140	0.145	0.150
Data collection			
Temperature (K)	123(2)	153(2)	153(2)
No. of collected reflections	24514	56454	60449
within the *Θ*-limit (°)	2.6–26.0	2.6–29.0	1.4–26.0
Index ranges ±*h*, ±*k*, ±*l*	-11/11, -13/14, -23/23	-12/12, -16/16, -25/25	-20/17, -35/35, -18/18
No. of unique reflections	6958	9660	14275
R_{int}	0.0514	0.0214	0.1138
Refinement calculations: full-matrix least- squares on all F^2 values			
Weighting expression *w* [a]	$[\sigma^2(F_o^2)+(0.1231P)^2 + 4.8205P]^{-1}$	$[\sigma^2(F_o^2)+(0.0698P)^2 + 0.8577P]^{-1}$	$[\sigma^2(F_o^2)+(0.0696P)^2 + 7.2667P]^{-1}$
No. of refined parameters	435	527	956
No. of F values used [$I>2\sigma(I)$]	5197	8789	9632
Final *R*-Indices			
$R(=\Sigma\|\Delta F\| / \Sigma\|F_o\|)$	0.0899	0.0445	0.0659
wR on F^2	0.2624	0.1258	0.1739
S (=Goodness of fit on F^2)	1.035	1.032	1.069
Final $\Delta\rho_{max}/\Delta\rho_{min}$ (e $Å^{-3}$)	0.74/-0.83	0.55/-0.47	0.58/-0.45

[a] $P = (F_o^2 + 2F_c^2)/3$

Table S1. Continued

Compound	**4S**	**5S**	**6S**
Empirical formula	$C_{34}H_{44}N_4 \cdot 2\ PF_6 \cdot H_2O$	$C_{33}H_{43}N_5 \cdot 2\ PF_6 \cdot C_2H_6O$	$C_{33}H_{45}N_7 \cdot 2\ PF_6 \cdot CHCl_3$
Formula weight	816.68	845.73	949.07
Crystal system	Triclinic	Triclinic	Triclinic
Space group	*P*-1	*P*-1	*P*-1
a (Å)	10.2169(9)	8.855(10)	9.982(4)
b (Å)	11.0273(11)	12.011(13)	12.256(5)
c (Å)	18.0807(17)	20.29(3)	18.045(10)
α(°)	101.363(8)	86.14(9)	92.52(4)
β(°)	94.747(7)	78.31(9)	99.47(4)
γ (°)	109.207(7)	70.52(8)	100.00(3)
V (Å^3)	1861.9(3)	1992(4)	2138.5(17)
Z	2	2	2
F(000)	848	880	976
D_c (Mg m^{-3})	1.457	1.410	1.474
μ (mm^{-1})	0.211	0.201	0.377
Data collection			
Temperature (K)	123(2)	143(2)	203(2)
No. of collected reflections	33943	19590	29887
within the Θ-limit (°)	2.7–26.5	2.7–28.3	2.6–27.1
Index ranges $\pm h$, $\pm k$, $\pm l$	-12/12, -13/11, -22/22	-11/11, -13/15, -26/26	-12/12, -15/15, -23/23
No. of unique reflections	7704	9734	9382
R_{int}	0.0467	0.0290	0.0312
Refinement calculations: full-matrix least- squares on all F^2 values			
Weighting expression w [a]	$[\sigma^2(F_o^2)+(0.0228P)^2 + 1.5566P]^{-1}$	$[\sigma^2(F_o^2)+(0.0404P)^2 + 1.3320P]^{-1}$	$[\sigma^2(F_o^2)+(0.0941P)^2 + 2.1657P]^{-1}$
No. of refined parameters	555	601	644
No. of F values used [I>2σ(I)]	6076	7227	7851
Final R-Indices			
$R(=\Sigma\|\Delta F\| / \Sigma\|F_o\|)$	0.0394	0.0414	0.0611
wR on F^2	0.0894	0.1117	0.1825
S (=Goodness of fit on F^2)	1.037	1.036	1.021
Final $\Delta\rho_{max}/\Delta\rho_{min}$ (e Å^{-3})	0.35/-0.27	0.33/-0.29	0.96/-0.97

[a] $P = (F_o^2 + 2F_c^2)/3$

Table S2. Relevant conformational parameters of the crystal structures of **1a**, **2S** (**2a**•EtOH), **3S** [**3a**•MeOH•H_2O (2:1:3)], **4S** (**4a**•H_2O), **5S** (**5a**•EtOH) and **6S** (**6a**•$CHCl_3$).

Compound	**1a**	**2S**	**3S**	**4S**	**5S**	**6S**
Dihedral angles (°)[a]						
mpla(A)-mpla(B)	75.5(1)	87.5(1)	82.8(3)	78.7(1)	75.4(1)	80.7(1)
mpla(A)-mpla(C)	81.8(1)	73.6(1)	89.6(3)	89.2(1)	80.6(1)	88.9(1)
mpla(A)-mpla(D)	89.5(1)	87.3(1)	84.4(3)	80.1(1)	84.4(1)	85.2(1)
mpla(B)-mpla(C)	52.3(1)	19.2(1)	37.9(3)	79.7(1)	51.8(1)	65.4(1)
mpla(B)-mpla(D)	82.5(1)	83.1(1)	62.8(3)	19.3(1)	78.9(1)	68.7(1)
mpla(C)-mpla(D)	50.9(1)	87.5(1)	81.0(3)	81.0(1)	36.8(1)	46.1(1)
mpla(A')-mpla(B')			82.4(3)			
mpla(A')-mpla(C')			88.2(3)			
mpla(A')-mpla(D')			79.8(3)			
mpla(B')-mpla(C')			42.3(3)			
mpla(B')-mpla(D')			48.4(3)			
mpla(C')-mpla(D')			89.5(3)			
Torsion angles						
C1-C7-N1-C8	-156.5(3)	-160.7(1)	-171.0(5)	156.9(2)	-160.1(2)	-173.5(2)
C7-N1-C8-N3					173.2(2)	-177.5(3)
C7-N1-C8-N2	164.8(3)	176.6(1)		-11.9(2)		
C3-C15-N2-C16			166.3(6)			
C3-C16-N4-C17		137.9(1)			-173.4(2)	-176.7(2)
C3-C17-N3-C18	-170.3(3)			173.5(1)		
C16-N4-C17-N5		-10.0(2)				
C17-N3-C18-N4	-14.7(5)					
C5-C24-N5-C25			-174.4(6)			
C5-C25-N5-C26					159.8(1)	
C5-C25-N6-C26						163.3(2)
C5-C25-N7-C26		162.1(1)				
C5-C26-N4-C27				-163.4(1)		
C5-C27-N5-C28	177.6(3)					
C15-N2-C16-N3			2.1(9)			
C24-N5-C25-N6			0.4(1)			
C1A-C7A-N1A-C8A			-169.1(6)			
C3A-C15A-N2A-C16A			-154.4(6)			
C5A-C24A-N5A-C25A			179.4(6)			
C15A-N2A-C16A-N3A			1.3(9)			
C24A-N5A-C25A-N6A			-4.2(9)			

[a] mpla means least-squares plane through the aromatic ring.

1a: Ring A: C1…C6; ring B: N2,C8…C12; ring C: N4,C18…C22; ring D: N5,C28…C32.

2S: Ring A: C1…C6; ring B: N2,N3,C8…C11; ring C: N5,N6,C17…C20; ring D: N7,C26…C30.

3S: Ring A: C1…C6; ring B: N1,C8…C12; ring C: N3,N4,C16…C19; ring D: N6,N7,C25…C28; Ring A’: C1A…C6A; ring B’: N1A,C8A…C12A; ring C’: N3A,N4A,C16A…C19A; ring D’: N6A,N7A,C25A…C28A.
4S: Ring A: C1…C6; ring B: N2,C8…C12; ring C: N3,C18…C22; ring D: N4,C27…C31.
5S: Ring A: C1…C6; ring B: N2,N3,C8…C11; ring C: N4,C17…C21; ring D: N5,C26…C30.
6S: Ring A: C1…C6; ring B: N2,N3,C8…C11; ring C: N4,C17…C21; ring D: N6,C26…C30.

Table S3. Geometric parameters for non-covalent interactions in the crystal structures examined.

Atoms		Distance		Angle	Figure
D-H···A		D···A	H···A	D-H···A	
P-F···π		P···*Cg*	F···*Cg*	P-F···*Cg*	
C-Cl···π		C···*Cg*	Cl···*Cg*	C-Cl···*Cg*	
π ···π		*Cg*···*Cg*			
**1a**					
N(1)-H(1)···F(3)	*x*, *y*, *z*	3.007(6)	2.29(6)	142(5)	**a** Fig.5
N(1)-H(1)···F(6A)	*x*, *y*, *z*	3.275(5)	2.43(6)	175(4)	
N(3)-H(3)···F(2)	*x*, *y*, *z*	3.319(5)	2.54(4)	151(4)	**b** Fig.5
N(3)-H(3)···F(2A)	*x*, *y*, *z*	2.829(5)	1.97(4)	169(4)	
N(3)-H(3)···F(5)	*x*, *y*, *z*	3.399(7)	2.58(4)	159(4)	**c** Fig.5
C(28)-H(28)···N(2)	1+*x*, *y*, *z*	3.385(6)	2.57	144	
C(30)-H(30)···F(4)	1-*x*, 2-*y*, 2-*z*	3.282(6)	2.37	160	**d** Fig.5
C(32)-H(32)···F(2)	*x*, *y*, *z*	3.234(6)	2.36	153	**e** Fig.5
C(34)-H(34A)···N(1)	*x*, *y*, *z*	3.322(6)	2.60	130	
C(33)-H(33B)···*Cg*(B)[a]	1-*x*, 1-*y*, 2-*z*	3.398(6)	2.80	120	
P(1)-F(2)···*Cg*(A)[a]	*x*, *y*, *z*	4.597(2)	3.199(4)	144.9(2)	
Cg(C)···*Cg*(C)[a]	1-*x*, 2-*y*, 1-*z*	3.862(2)			**f** Fig.5
**2S**					
N(1)-H(1)···F(3)	1+*x*, *y*, *z*	3.085(2)	2.30(2)	148(2)	**a** Fig.6b
N(1)-H(1)···F(5)	1+*x*, *y*, *z*	3.322(2)	2.52(1)	151(2)	
N(1)-H(1)···F(5A)	1+*x*, *y*, *z*	3.027(8)	2.14(1)	172(2)	
N(4)-H(4)···F(2)	1+*x*, *y*, *z*	3.201(2)	2.46(1)	142(2)	**b** Fig.6b
O(1B)-H(1B)···N(6)	-1+*x*, *y*, *z*	2.884(2)	2.02(1)	176(2)	**c** Fig.6b
C(1B)-H(1B1)···F(6A)	*x*, *y*, *z*	3.183(8)	2.40	135	
C(13)-H(13A)···N(6)	1+*x*, 1+*y*, *z*	3.461(2)	2.62	144	
C(13)-H(13B)···F(4)	2+*x*, 1+*y*, *z*	3.393(2)	2.48	156	
C(21)-H(21B)···O(1B)	*x*, *y*, *z*	3.500(2)	2.57	159	**d** Fig.6b
C(22)-H(22C)···N(3)	-1+*x*, -1+*y*, *z*	3.548(2)	2.68	148	
C(25)-H(25A)···F(3)	*x*, *y*, *z*	3.211(2)	2.46	133	
C(26)-H(26)···N(2)	-1+*x*, *y*, *z*	3.376(2)	2.48	157	
C(28)-H(28)···F(1A)	1-*x*, 1-*y*, 1-*z*	3.128(2)	2.36	138	
C(28)-H(28)···F(6)	1-*x*, 1-*y*, 1-*z*	3.286(2)	2.35	171	**e** Fig.6b
C(28)-H(28)···F(6A)	1-*x*, 1-*y*, 1-*z*	3.298(7)	2.39	161	
C(29)-H(29)···F(4A)	1+*x*, *y*, *z*	3.320(7)	2.61	132	
C(30)-H(30)···F(2A)	1+*x*, *y*, *z*	3.183(8)	2.40	145	
C(30)-H(30)···F(5)	1+*x*, *y*, *z*	3.063(2)	2.51	117	**f** Fig.6b
**3S**					
O(1)-H(1)···N(7A)	*x*, *y*, -1+*z*	2.690(7)	1.85	172	**a** Fig.7b
O(1A)-H(1A)···N(7)	*x*, *y*, 1+*z*	2.631(8)	1.81	163	**b** Fig.7b
O(1B)-H(1B)···N(4A)	1.5-*x*, 0.5+*y*, -0.5+*z*	2.724(9)	1.91	159	**c** Fig.7b

O(1W)-H(1W1)···N(4)	-*x*, 1-*y*, 0.5+z	2.873(9)	2.03	169	**d**	Fig.7b
O(1W)-H(1W2)···F(1)	0.5-*x*, 0.5+*y*, 0.5+z	3.078(9)	2.28	156	**e**	Fig.7b
O(1W)-H(1W2)···F(4)	0.5-*x*, 0.5+*y*, 0.5+z	3.228(9)	2.49	146	**f**	Fig.7b
O(1W)-H(1W2)···F(5)	0.5-*x*, 0.5+*y*, 0.5+z	3.198(9)	2.51	139	**g**	Fig.7b
O(2W)-H(2W1)···O(1W)	*x*, *y*, *z*	2.820(9)	2.02	157	**h**	Fig.7b
O(2W)-H(2W2)···O(1B)	-1+*x*, *y*, *z*	2.696(9)	1.86	168	**i**	Fig.7b
O(3W)-H(3W1)···O(2W)	0.5-*x*, -0.5+*y*, 0.5+z	2.817(8)	1.98	170	**j**	Fig.7b
O(3W)-H(3W2)···O(1)	*x*, *y*, 1+z	3.034(8)	2.49	122	**k**	Fig.7b
N(2)-H(2)···F(4)	-0.5+*x*, 0.5-*y*, *z*	3.193(7)	2.41	146	**l**	Fig.7b
N(5)-H(5)···F(3)	-0.5+*x*, 0.5-*y*, *z*	3.247(7)	2.59	131	**m**	Fig.7b
N(2A)-H(2A)···O(2W)	0.5-*x*, -0.5+*y*, 0.5+z	3.052(8)	2.27	145	**n**	Fig.7b
N(5A)-H(5A)···O(3W)	*x*, *y*, *z*	2.973(9)	2.18	146	**o**	Fig.7b
C(1B)-H(1B3)···F(5A)	*x*, *y*, *z*	3.462(15)	2.53	159	**p**	Fig.7b
C(7)-H(7A)···F(4B)	1-*x*, 1-*y*, -0.5+z	3.095(15)	2.27	140		
C(7)-H(7B)···F(6)	*x*, *y*, *z*	3.356(8)	2.51	144		
C(8)-H(8)···O(1A)	0.5+*x*, 0.5-*y*, -1+z	3.496(8)	2.56	169		
C(8A)-H(8A)···F(1A)	0.5-*x*, -0.5+*y*, 0.5+z	3.286(13)	2.36	165		
C(8A)-H(8A)···F(1B)	0.5-*x*, -0.5+*y*, 0.5+z	3.24(2)	2.35	157		
C(11A)-H(11A)···F(1)	*x*, *y*, 1+z	3.289(10)	2.41	154		
C(12)-H(12)···F(3)	-0.5+*x*, 0.5-*y*, *z*	3.183(9)	2.38	142		
C(12)-H(12)···F(5)	-0.5+*x*, 0.5-*y*, *z*	3.179(8)	2.50	129		
C(18A)-H(18A)···F(6A)	0.5+*x*, 0.5-*y*, *z*	3.325(14)	2.43	157		
C(18A)-H(18A)···F(6B)	0.5+*x*, 0.5-*y*, *z*	3.196(16)	2.25	172		
C(20)-H(20A)···N(3A)	-0.5+*x*, 0.5-*y*, *z*	3.584(10)	2.61	172		
C(27A)-H(27A)···F(3A)	*x*, *y*, *z*	3.403(13)	2.45	176		
C(27)-H(27)···F(3B)	-0.5+*x*, 0.5-*y*, *z*	3.43(2)	2.51	165		
C(29A)-H(29E)···F(6A)	*x*, *y*, *z*	3.398(13)	2.49	154		
C(30)-H(30B)···F(2A)	-0.5+*x*, 0.5-*y*, *z*	3.453(14)	2.48	171		
C(30)-H(30B)···F(2B)	-0.5+*x*, 0.5-*y*, *z*	3.46(2)	2.49	175		
C(30A)-H(30D)···O(1)	*x*, *y*, 1+z	3.323(8)	2.57	134		
C(30A)-H(30D)···F(1A)	1-*x*, 1-*y*, 0.5+z	3.297(13)	2.48	141		
C(21)-H(21B)···*Cg*(C')[a]	0.5-*x*, 0.5+*y*, -0.5+z	3.720(9)	2.93	138		
C(29)-H(29A)···*Cg*(A')[a]	*x*, *y*, *z*	3.473(10)	2.95	115		
C(29A)-H(29F)···*Cg*(A)[a]	*x*, *y*, *z*	3.526(9)	2.67	146		
P(1)-F(2)···*Cg*(B')[a]	0.5+*x*, 0.5-*y*, -1+z	4.346(4)	3.151(6)	130.5(3)		
P(1)-F(3)···*Cg*(A)[a]	0.5+*x*, 0.5-*y*, *z*	4.488(3)	3.252(5)	133.2(2)		
P(1A)-F(5A)···*Cg*(B)[a]	1-*x*, 1-*y*, 0.5+z	4.424(3)	3.065(9)	139.3(5)		
P(1A)-F(5B)···*Cg*(B)[a]	1-*x*, 1-*y*, 0.5+z	4.424(3)	3.227(9)	129.8(7)		
**4S**						
N(1)-H(1)···F(3A)	1-*x*, 1-*y*, 1-*z*	3.091(2)	2.21(1)	176(2)	**a**	Fig.10
O(1W)-H(1WA)···F(5A)	*x*, *y*, *z*	2.934(2)	2.08(2)	172(3)	**b**	Fig.10
O(1W)-H(1WA)···F(2B)	*x*, *y*, *z*	3.173(14)	2.46(3)	140(2)		
O(1W)-H(1WA)···F(5B)	*x*, *y*, *z*	3.076(11)	2.33(2)	145(3)		
O(1W)-H(1WB)···F(3)	1-*x*, 1-*y*, 1-*z*	2.911(2)	2.05(2)	173(3)	**c**	Fig.10
O(1W)-H(1WB)···F(6)	1-*x*, 1-*y*, 1-*z*	3.142(2)	2.54(3)	128(2)	**d**	Fig.10
C(12)-H(12)···F(1B)	1-*x*, 1-*y*, 1-*z*	3.293(8)	2.47	145		
C(17)-H(17A)···O(1W)	1-*x*, 2-*y*, 1-*z*	3.357(2)	2.48	149		

C(17)-H(17B)···F(1)	1-*x*, 1-*y*, -*z*	3.386(2)	2.53	144	**e** Fig.10
C(17)-H(17B)···F(5)	1-*x*, 1-*y*, -*z*	3.357(2)	2.54	140	**f** Fig.10
C(18)-H(18)···F(3)	1-*x*, 1-*y*, -*z*	3.390(2)	2.51	154	**g** Fig.10
C(21)-H(21)···F(4B)	1-*x*, 1-*y*, 1-*z*	3.247(10)	2.54	131	
C(22)-H(22)···F(2A)	1-*x*, 1-*y*, 1-*z*	3.149(2)	2.41	134	
C(22)-H(22)···F(6A)	1-*x*, 1-*y*, 1-*z*	3.174(2)	2.55	124	**h** Fig.10
C(22)-H(22)···F(6B)	1-*x*, 1-*y*, 1-*z*	3.201(11)	2.31	155	
C(25)-H(25C)···F(4A)	-*x*, 1-*y*, 1-*z*	3.421(3)	2.51	155	**i** Fig.10
C(25)-H(25C)···F(4B)	-*x*, 1-*y*, 1-*z*	3.476(11)	2.51	164	
C(26)-H(26B) ···F(6)	-1+*x*, *y*, *z*	3.439(2)	2.59	144	
C(27)-H(27)···F(6)	-1+*x*, *y*, *z*	3.118(2)	2.52	121	
C(29)-H(29)···F(5)	1-*x*, -*y*, -*z*	3.060(2)	2.53	115	**j** Fig.10
C(30)-H(30)···F(2B)	1-*x*, 1-*y*, 1-*z*	3.138(13)	2.54	121	
C(31)-H(31)···F(2A)	1-*x*, 1-*y*, 1-*z*	3.012(2)	2.39	123	**k** Fig.10
C(31)-H(31)···F(6B)	1-*x*, 1-*y*, 1-*z*	3.454(16)	2.58	154	
C(34)-H(34C)···F(1A)	*x*, *y*, *z*	3.402(3)	2.48	157	**l** Fig.10
C(34)-H(34C)···F(1B)	*x*, *y*, *z*	3.300(8)	2.38	157	
C(13)-H(13B)···*Cg*(A)[a]	1-*x*, 2-*y*, 1-*z*	3.609(2)	2.96	125	
P(1B)-F(6B)···*Cg*(A)[a]	1-*x*, 1-*y*, 1-*z*	4.804(10)	3.245(16)	167.2(9)	
Cg(B)···*Cg*(B)[a]	2-*x*, 2-*y*, 1-*z*	3.835(1)			
Cg(B)···*Cg*(D)[a]	1-*x*, 1-*y*, 1-*z*	3.955(2)			
Cg(C)···*Cg*(C)[a]	1-*x*, 1-*y*, -*z*	4.186(1)			**m** Fig.10
**5S**					
N(1)-H(1)···F(2B)	*x*, *y*, *z*	3.128(5)	2.29(2)	171(3)	**a** Fig.11
O(1A)-H(1A)···N(2)	1+*x*, -1+*y*, *z*	3.298(8)	2.49	159	**b** Fig.11
O(1AA)-H(1AA)···F(4)	1+*x*, *y*, *z*	3.043(8)	2.22	166	
C(12)-H(12A)···F(2A)	*x*, 1+*y*, *z*	3.369(11)	2.43	161	
C(12)-H(12A)···F(2)	*x*, 1+*y*, *z*	3.351(17)	2.43	157	**c** Fig.11
C(14)-H(14A)···N(4)	*x*, *y*, *z*	3.346(5)	2.60	133	
C(15)-H(15C)···F(4A)	*x*, 1+*y*, *z*	3.290(9)	2.48	140	
C(16)-H(16A)···F(3A)	1+*x*, *y*, *z*	3.350(8)	2.50	144	
C(17)-H(17)···F(3A)	1-*x*, 1-*y*, 1-*z*	3.278(9)	2.33	175	**d** Fig.11
C(17)-H(17)···F(3)	1-*x*, 1-*y*, 1-*z*	3.297(11)	2.38	161	
C(20)-H(20)···F(1B)	*x*, *y*, *z*	3.217(5)	2.48	135	**e** Fig.11
C(21)-H(21)···F(3B)	*x*, *y*, *z*	3.215(5)	2.30	161	**f** Fig.11
C(22)-H(22B)···F(1A)	*x*, *y*, *z*	3.404(11)	2.55	146	
C(26)-H(26)···N(3)	1+*x*, *y*, *z*	3.286(5)	2.38	159	
C(28)-H(28)···F(1B)	2-*x*, 1-*y*, -*z*	3.449(6)	2.51	168	**g** Fig.11
C(28)-H(28)···F(4B)	2-*x*, 1-*y*, -*z*	3.289(5)	2.54	136	**h** Fig.11
C(30)-H(30)···F(3B)	*x*, *y*, *z*	3.040(5)	2.24	142	
C(32)-H(32A)···N(1)	*x*, *y*, *z*	3.248(5)	2.52	130	
C(1A)-H(1A2)···F(4)	1+*x*, *y*, *z*	2.83(3)	2.35	109	
C(1AA)-H(1A3)···*Cg*(A)[a]	*x*, -1+*y*, *z*	3.848(10)	2.87	171	
**6S**					
N(1)-H(1)···F(3A)	*x*, *y*, *z*	3.141(10)	2.26(2)	174(2)	**a** Fig.12
N(1)-H(1)···F(3AA)	*x*, *y*, *z*	3.352(10)	2.54(2)	153(2)	

N(5)-H(5A)···F(4B)	1+*x*, -1+*y*, *z*	3.034(10)	2.41	128	
N(5)-H(5A)···F(1BA)	1+*x*, -1+*y*, *z*	3.209(10)	2.34	167	
N(5)-H(5B)···F(6A)	*x*, -1+*y*, *z*	2.922(8)	2.06	165	**b** Fig.12
N(5)-H(5B)···F(6AA)	*x*, -1+*y*, *z*	2.893(11)	2.03	167	
N(7)-H(7A)···F(2B)	1-*x*, 1-*y*, 1-*z*	3.285(7)	2.66	129	**c** Fig.12
N(7)-H(7B)···F(1A)	2-*x*, 1-*y*, 1-*z*	3.033(8)	2.22	154	**d** Fig.12
N(7)-H(7B)···F(1AA)	2-*x*, 1-*y*, 1-*z*	2.939(8)	2.06	174	
C(1A)-H(1A)···N(3)	*x*, *y*, *z*	3.335(4)	2.63	128	**e** Fig.12
C(12)-H(12B) ···F(1BA)	1-*x*, 1-*y*, -*z*	3.367(6)	2.54	142	
C(16)-H(16B)···F(1B)	1+*x*, -1+*y*, *z*	3.046(8)	2.54	111	
C(16)-H(16B)···F(6B)	1+*x*, -1+*y*, *z*	3.447(8)	2.48	164	
C(16)-H(16B)···F(6BA)	1+*x*, -1+*y*, *z*	3.408(9)	2.44	166	
C(19)-H(19)···F(1B)	*x*, -1+*y*, *z*	3.474(8)	2.66	144	
C(19)-H(19)···F(5BA)	*x*, -1+*y*, *z*	3.063(9)	2.51	117	
C(21)-H(21)···F(5A)	*x*, *y*, *z*	3.55(1)	2.71	149	**f** Fig.12
C(21)-H(21)···F(5AA)	*x*, *y*, *z*	3.515(11)	2.60	161	
C(22)-H(22A)···F(2A)	*x*, *y*, *z*	3.389(8)	2.46	158	
C(23)-H(23A)···F(6B)	1+*x*, -1+*y*, *z*	3.550(8)	2.56	176	
C(23)-H(23A)···F(6BA)	1+*x*, -1+*y*, *z*	3.421(11)	2.43	177	
C(25)-H(25B)···F(2BA)	1-*x*, 1-*y*, 1-*z*	3.439(13)	2.55	150	
C(27)-H(27)···F(4A)	2-*x*, 1-*y*, 1-*z*	3.388(8)	2.53	151	
C(27)-H(27)···F(6AA)	2-*x*, 1-*y*, 1-*z*	3.333(11)	2.52	143	
C(28)-H(28)···F(5B)	2-*x*, 1-*y*, 1-*z*	3.398(7)	2.50	159	
C(31)-H(31B)···F(3B)	2-*x*, 1-*y*, 1-*z*	3.444(7)	2.60	144	
C(31)-H(31C)···F(4AA)	1+*x*, *y*, *z*	3.168(11)	2.39	136	
C(32)-H(32A)···F(4BA)	1+*x*, *y*, *z*	3.380(13)	2.49	149	
C(33)-H(33B)···F(5BA)	1+*x*, *y*, *z*	3.281(11)	2.47	140	
C(33)-H(33C)···F(2A)	1+*x*, *y*, *z*	3.285(8)	2.55	132	
Cg(B)···*Cg*(B)[a]	2-*x*, 1-*y*, -*z*	3.629(3)			**g** Fig.12
C(1A)-Cl(2)···*Cg*(C)[a]	1-*x*, -*y*, -*z*		3.264(2)	172.0(1)	

[a] *Cg* means the centroid (centre of gravity) of the aromatic ring.

1a: Ring A: C(1)…C(6); ring B: N(2),C(8)…C(12); ring C: N(4),C(18)…C(22);

3S: Ring A: C(1)…C(6); ring B: N(1),C(8)…C(12); Ring A': C(1A)…C(6A); ring B': N(1A),C(8A)…C(12A); ring C': N(3A),N(4A),C(16A)…C(19A).

4S: Ring A: C(1)…C(6); ring B: N(2),C(8)…C(12); ring C: N(3),C(18)…C(22); ring D: N(4),C(27)…C(31).

5S: Ring A: C(1)…C(6).

6S: Ring B: N(2),N(3),C(8)…C(11); ring C: N4,C17…C21.

Superposition of the two receptor cations observed in the crystal structure 3S (Figure S1)

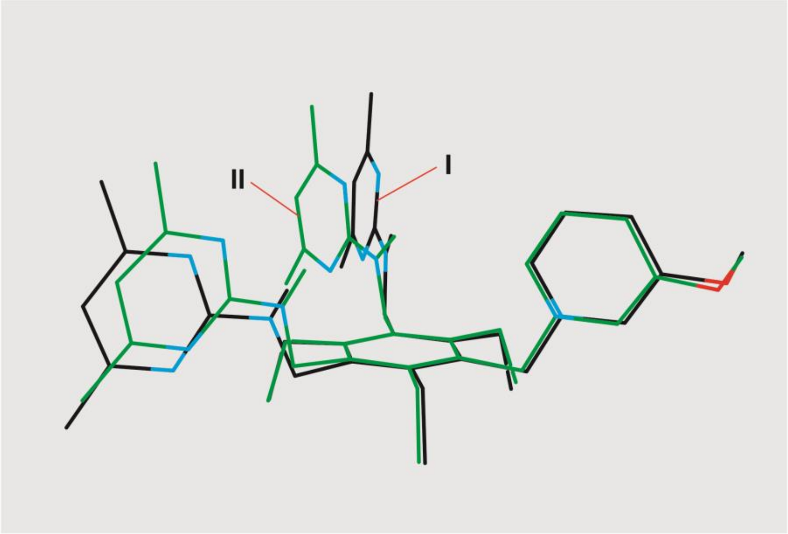

Figure S1. Superposition of the two receptor cations found in the crystal structure **3S** [**3a**•MeOH•H_2O (2:1:3)]. The $\mathbf{3}^+$ cation of complex I [**3S-I**; $\mathbf{3}^+PF_6^-$•H_2O] is highlighted in black, that of complex II [**3S-II**; $\mathbf{3}^+PF_6^-$•$(H_2O)_2$•CH_3OH] in green; nitrogen atoms are colored blue, oxygen atoms in red.

Dihedral angles between pairs of identical arene units (A/A', B/B' and C/C') are: A/A' = 20.2(3)°; B/B' = 11.4(4)° and C/C' = 3.3(4)° [for designation of the aromatic rings, see Figure 3 in the manuscript].

^{1}H and ^{13}C NMR spectra of compounds 1a–6a and 1b–6b (Figures S2a-S13a and S2b-S13b).

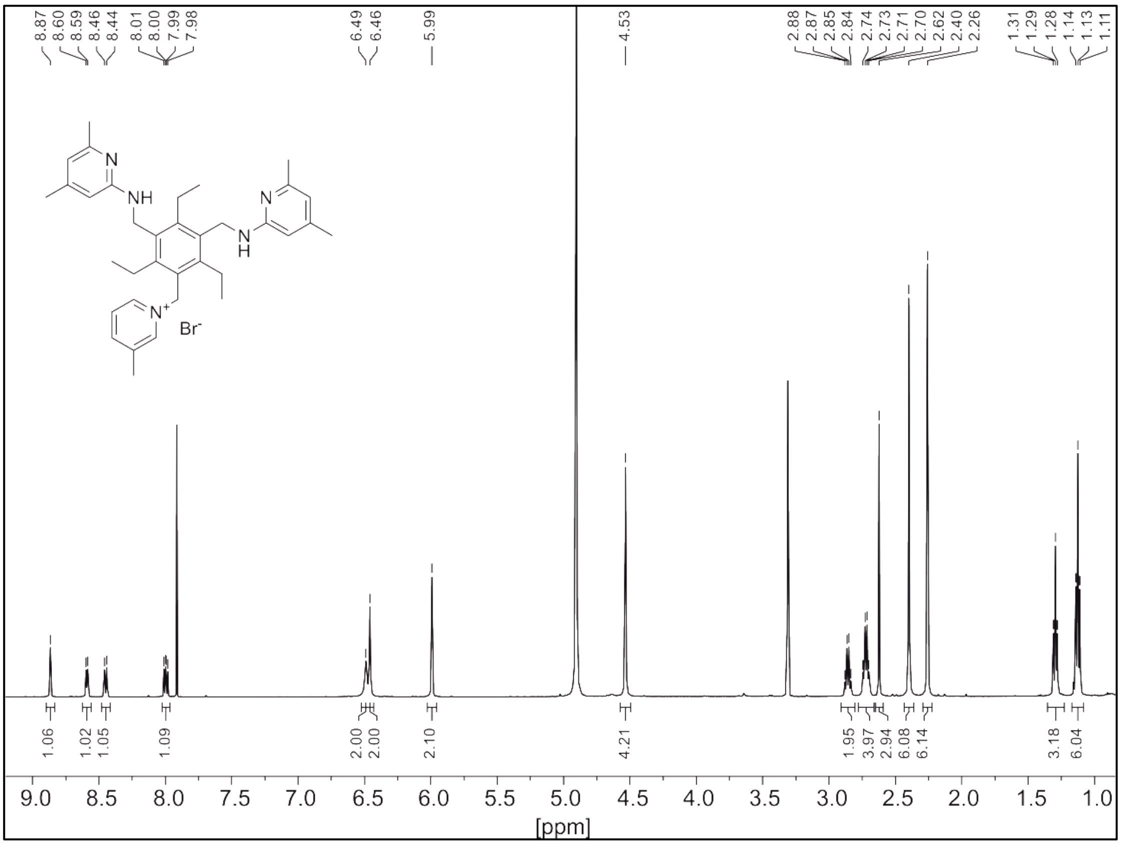

Figure S2a. ^{1}H NMR (500 MHz) spectrum of **1b** in CD_3OD.

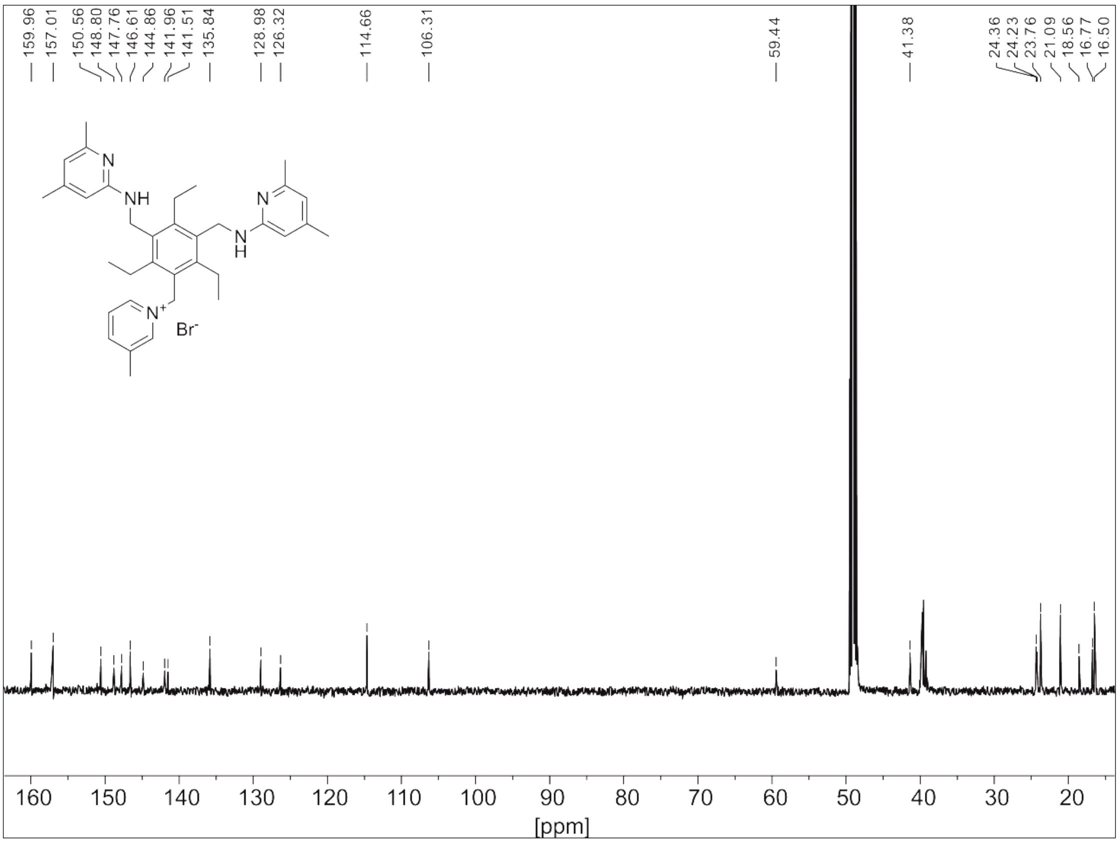

Figure S2b. ^{13}C NMR (125 MHz) spectrum of **1b** in CD_3OD/DMSO-d_6 (5:2, v/v).

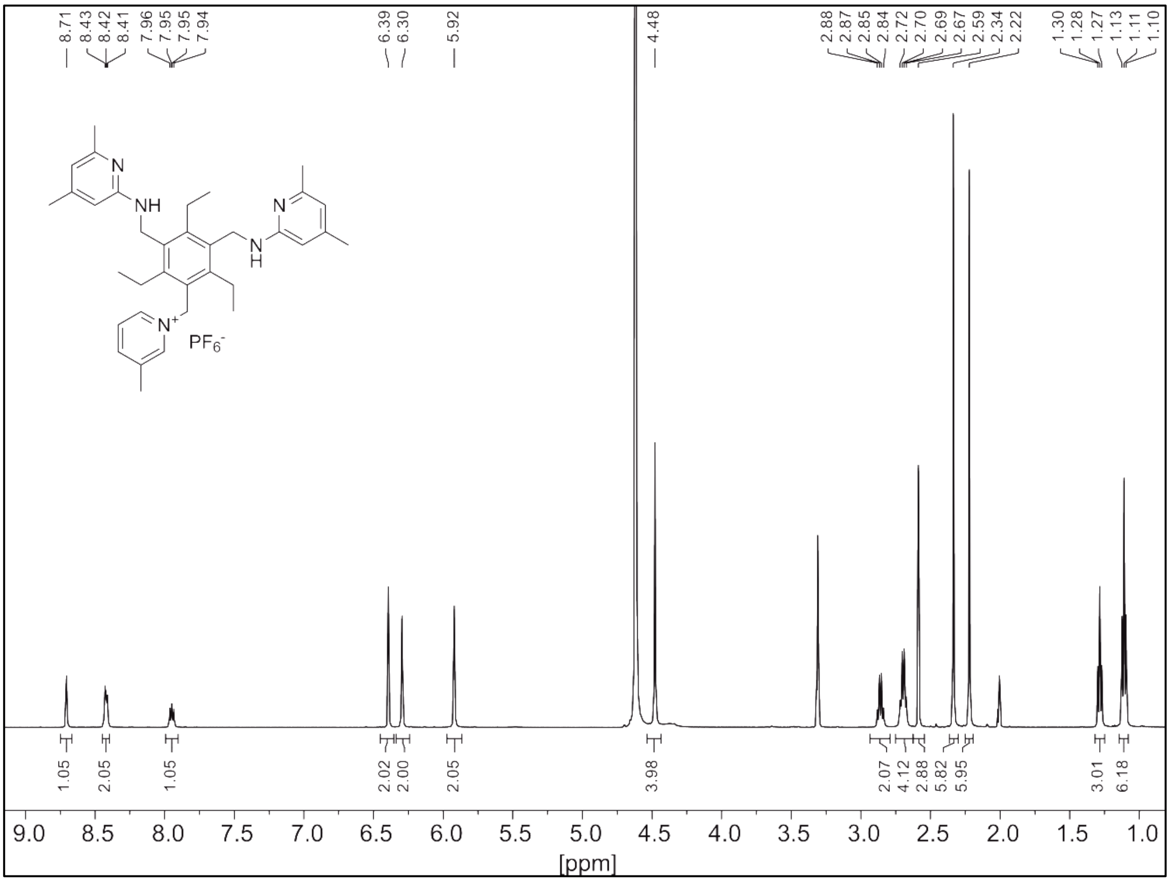

Figure S3a. ^{1}H NMR (500 MHz) spectrum of **1a** in CD_3OD/CD_3CN (6:0.1, v/v).

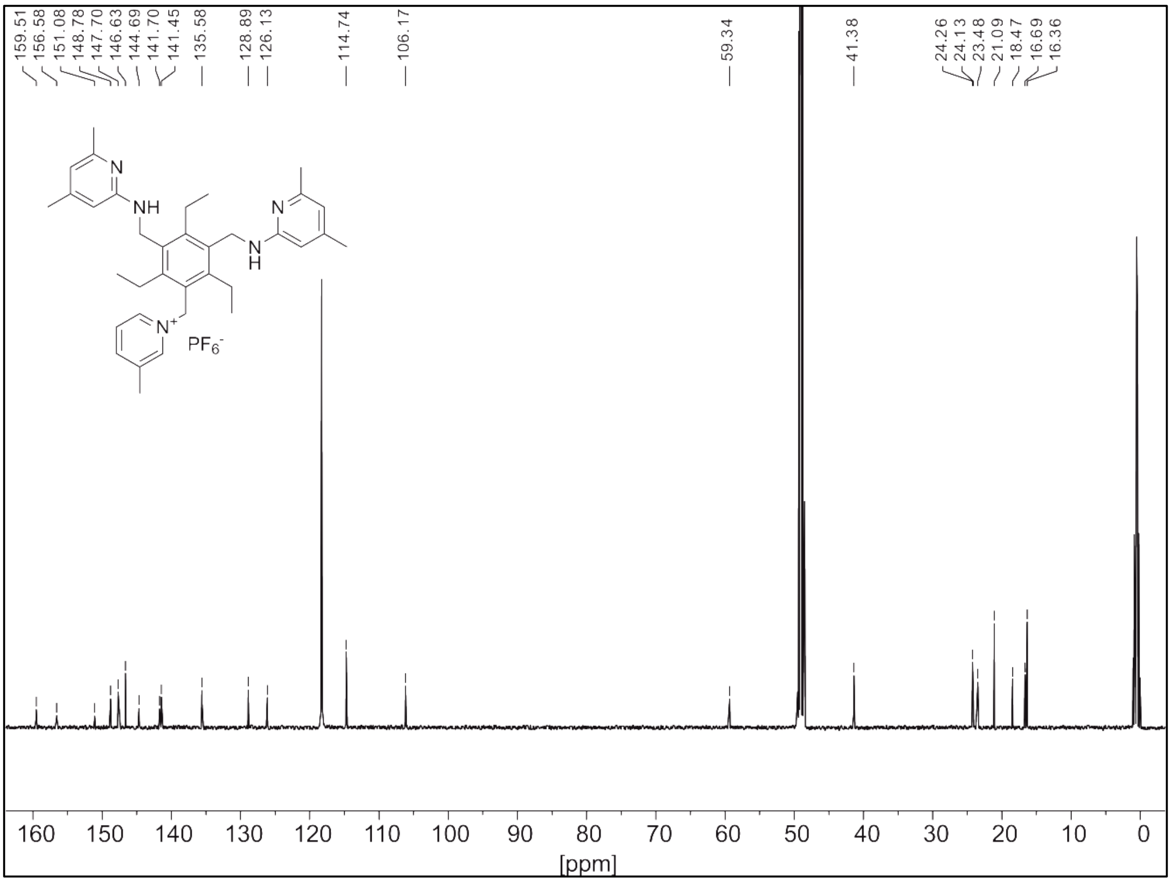

Figure S3b. ^{13}C NMR (125 MHz) spectrum of **1a** in CD_3OD/CD_3CN (6:0.1, v/v).

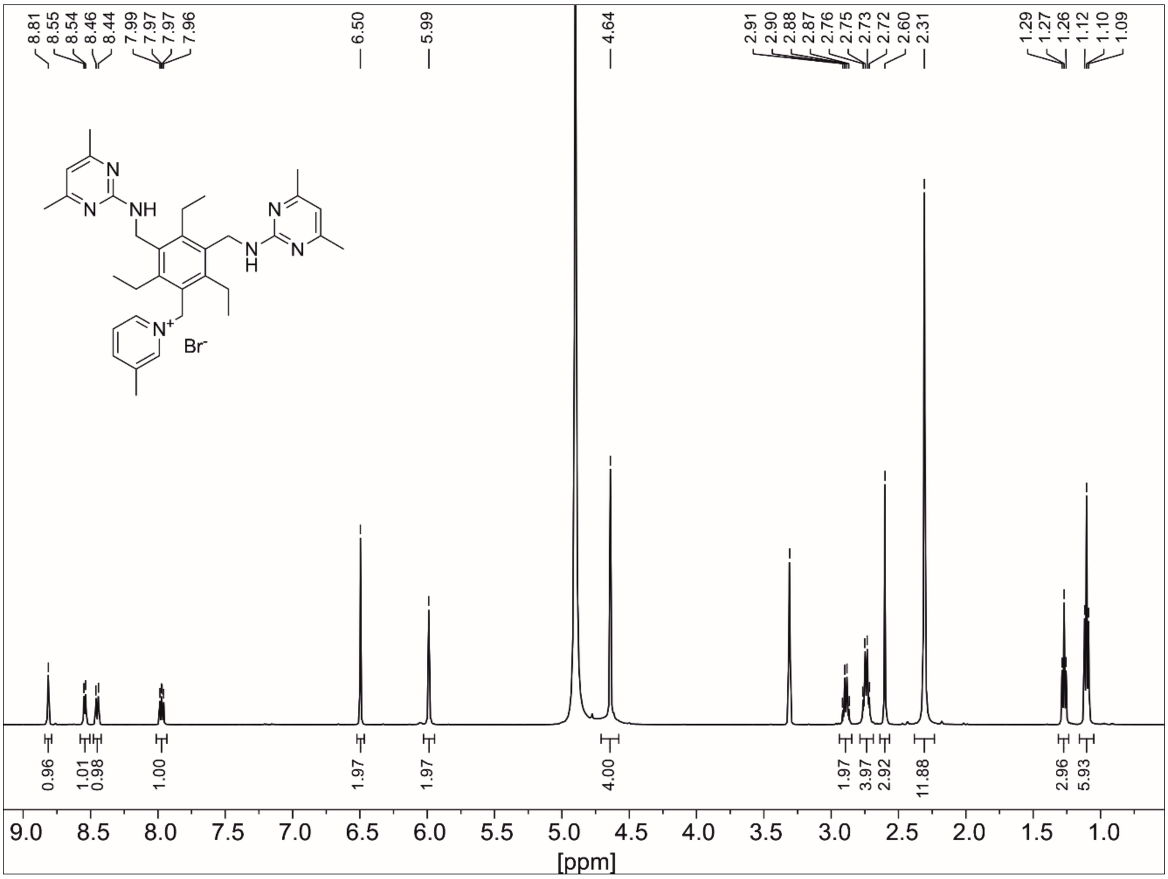

Figure S4a. ^{1}H NMR (500 MHz) spectrum of **2b** in CD_3OD.

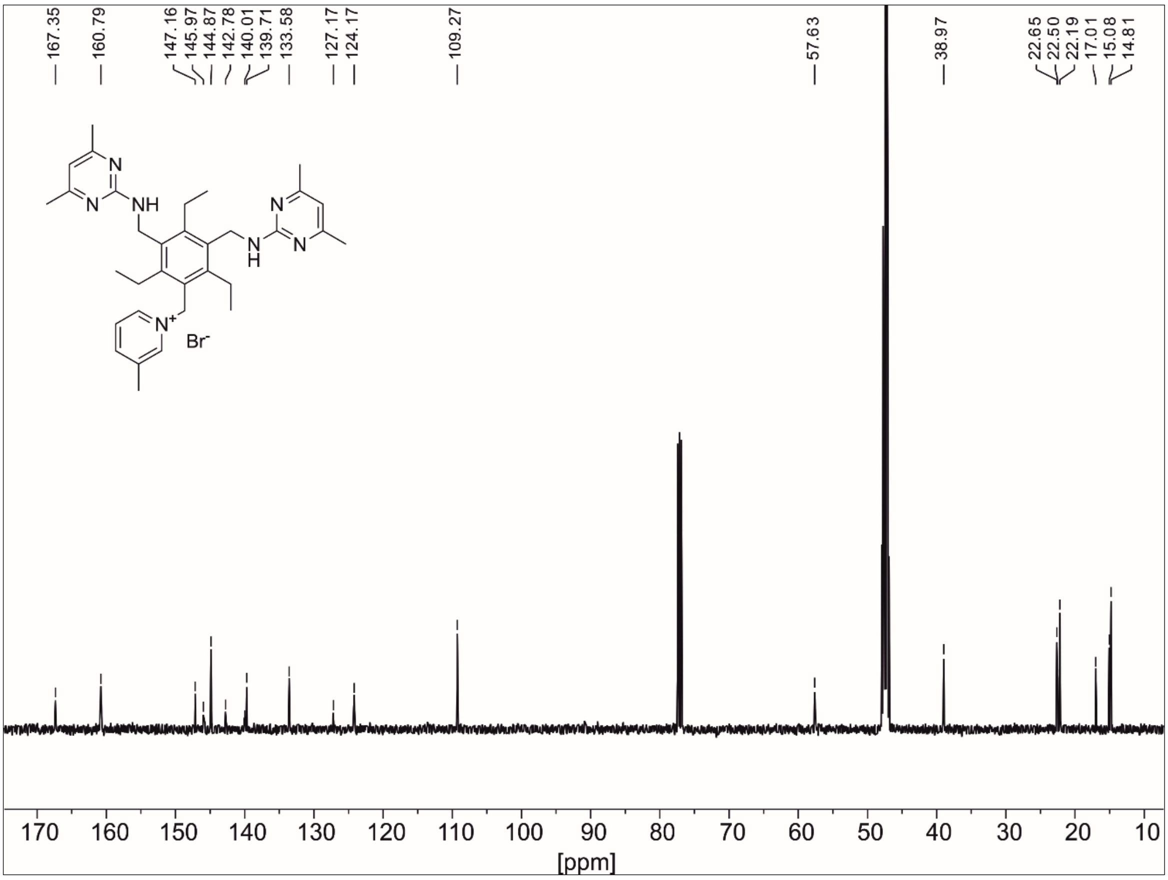

Figure S4b. ^{13}C NMR (125 MHz) spectrum of **2b** in $CD_3OD/CDCl_3$ (5:2, v/v).

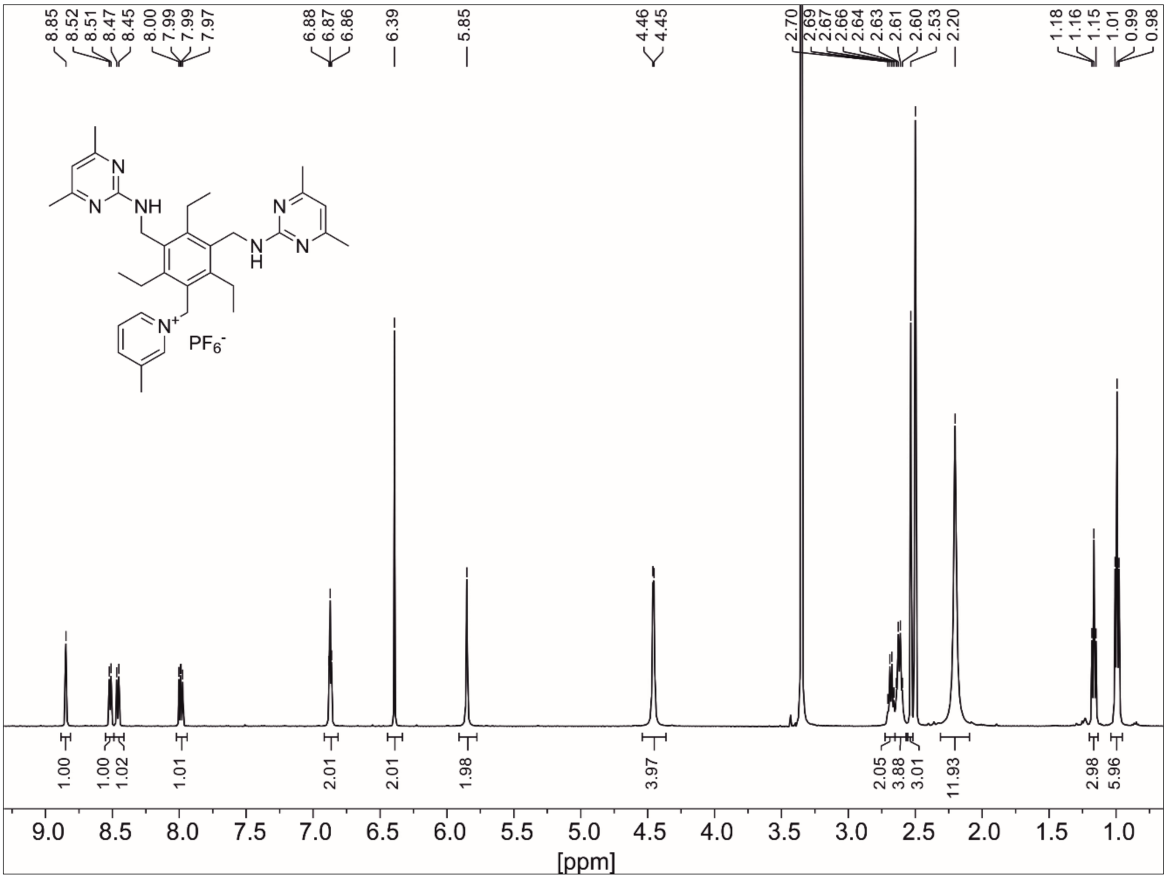

Figure S5a. ^{1}H NMR (500 MHz) spectrum of **2a** in DMSO-d_6.

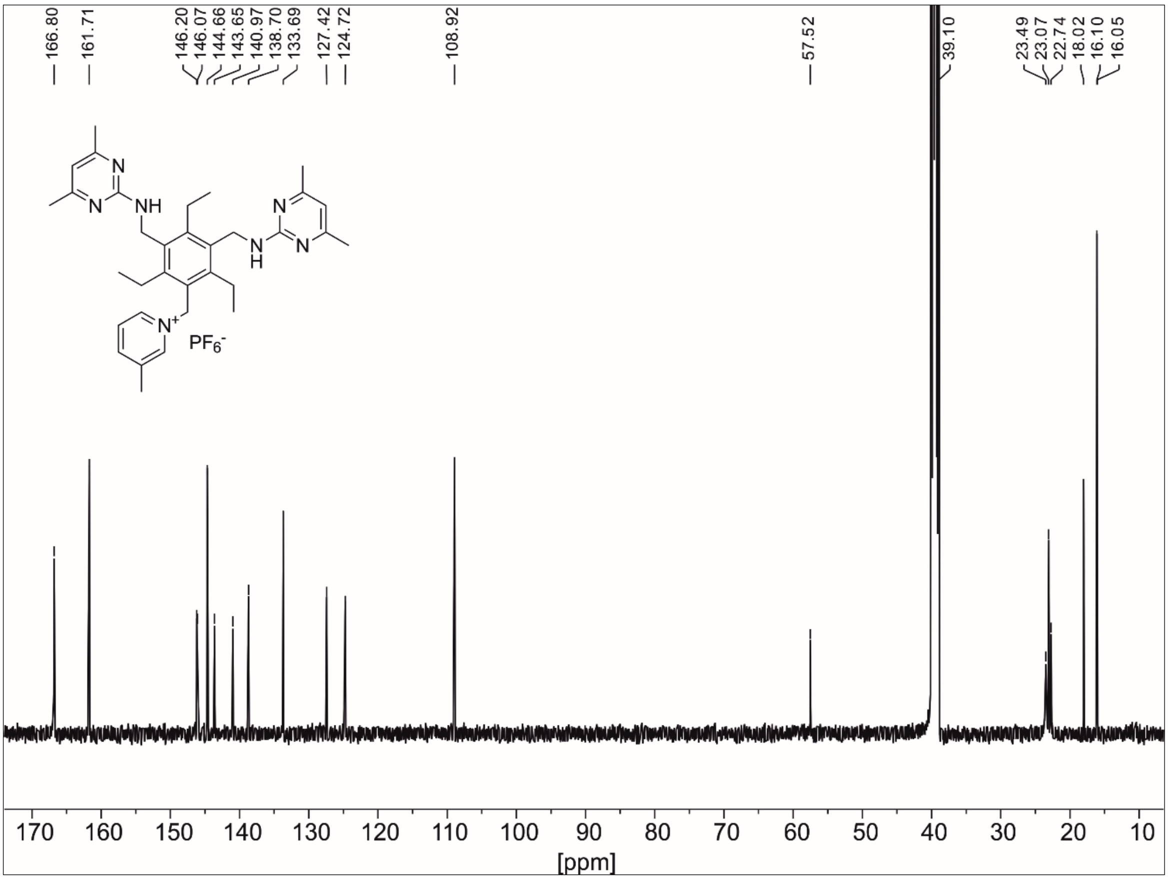

Figure S5b. ^{13}C NMR (125 MHz) spectrum of **2a** in DMSO-d_6.

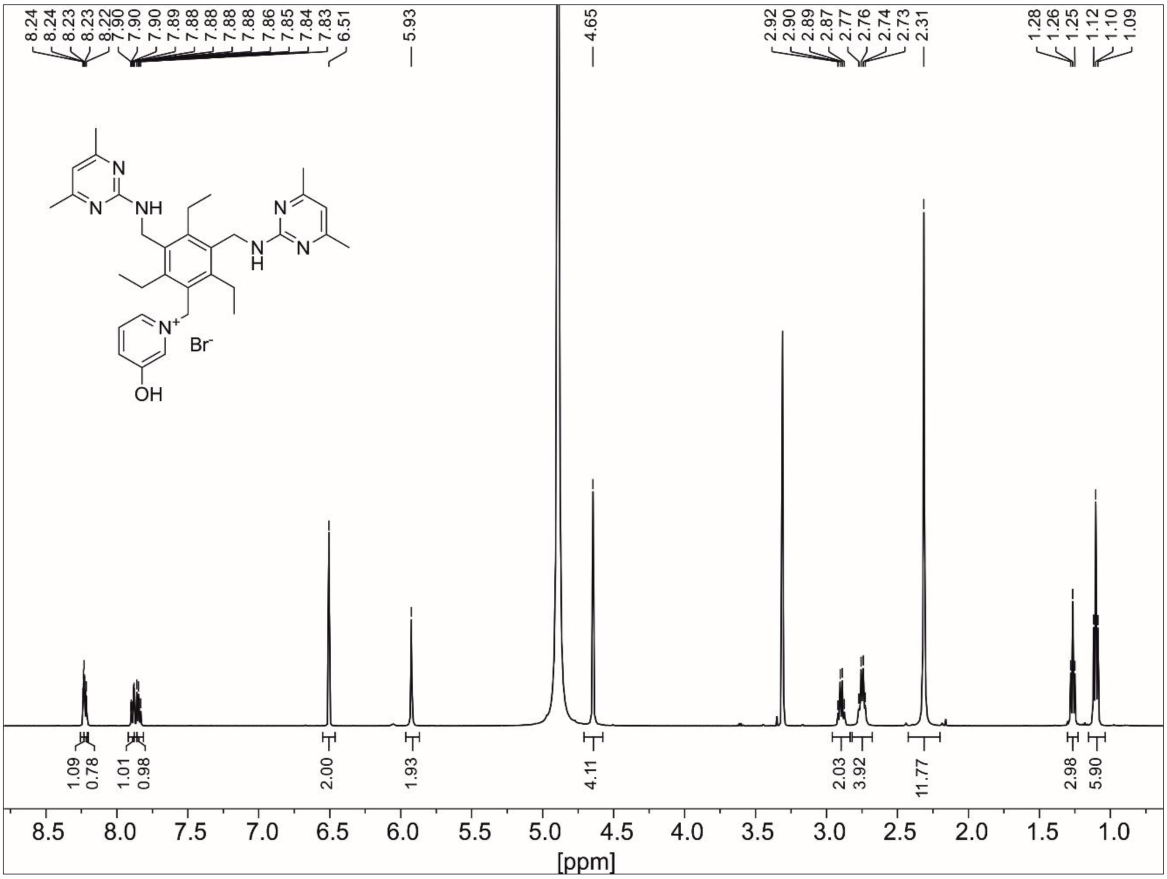

Figure S6a. ^{1}H NMR (500 MHz) spectrum of **3b** in CD_3OD.

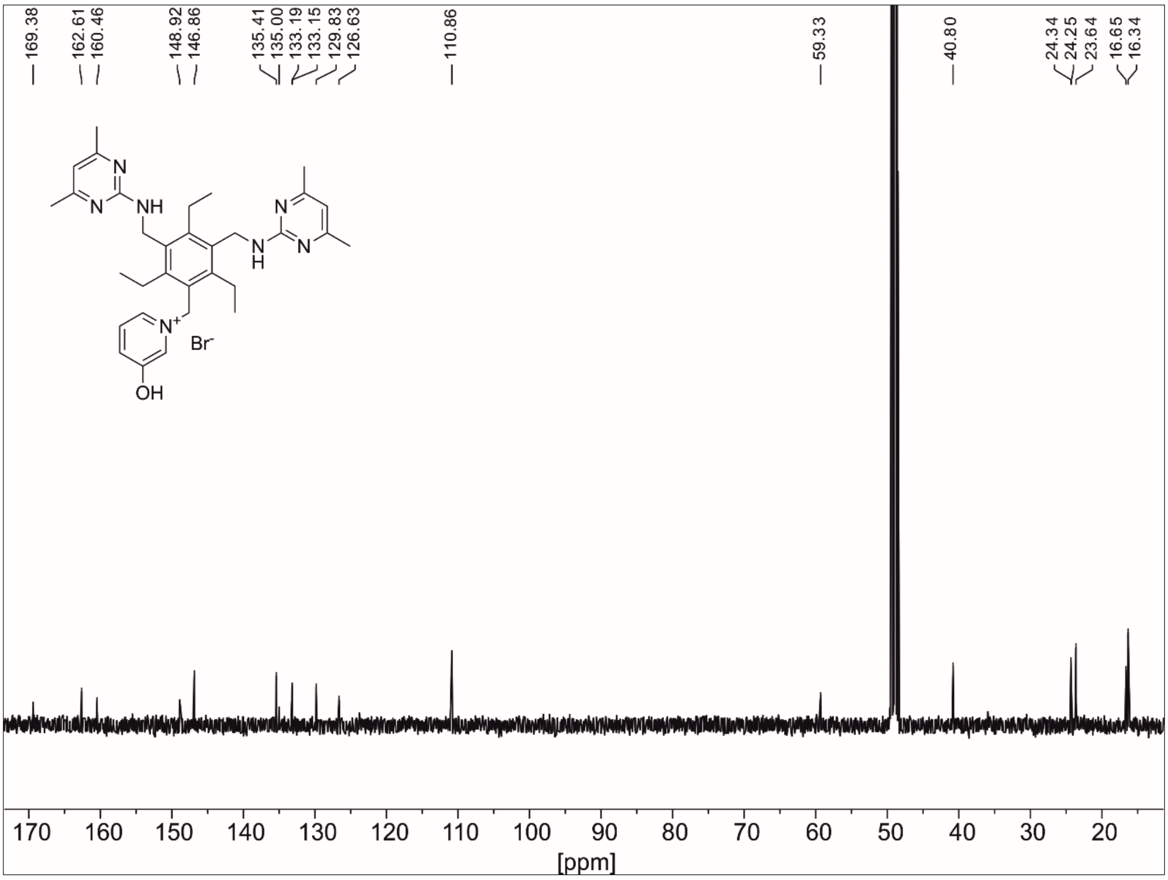

Figure S6b. ^{13}C NMR (125 MHz) spectrum of **3b** in CD_3OD.

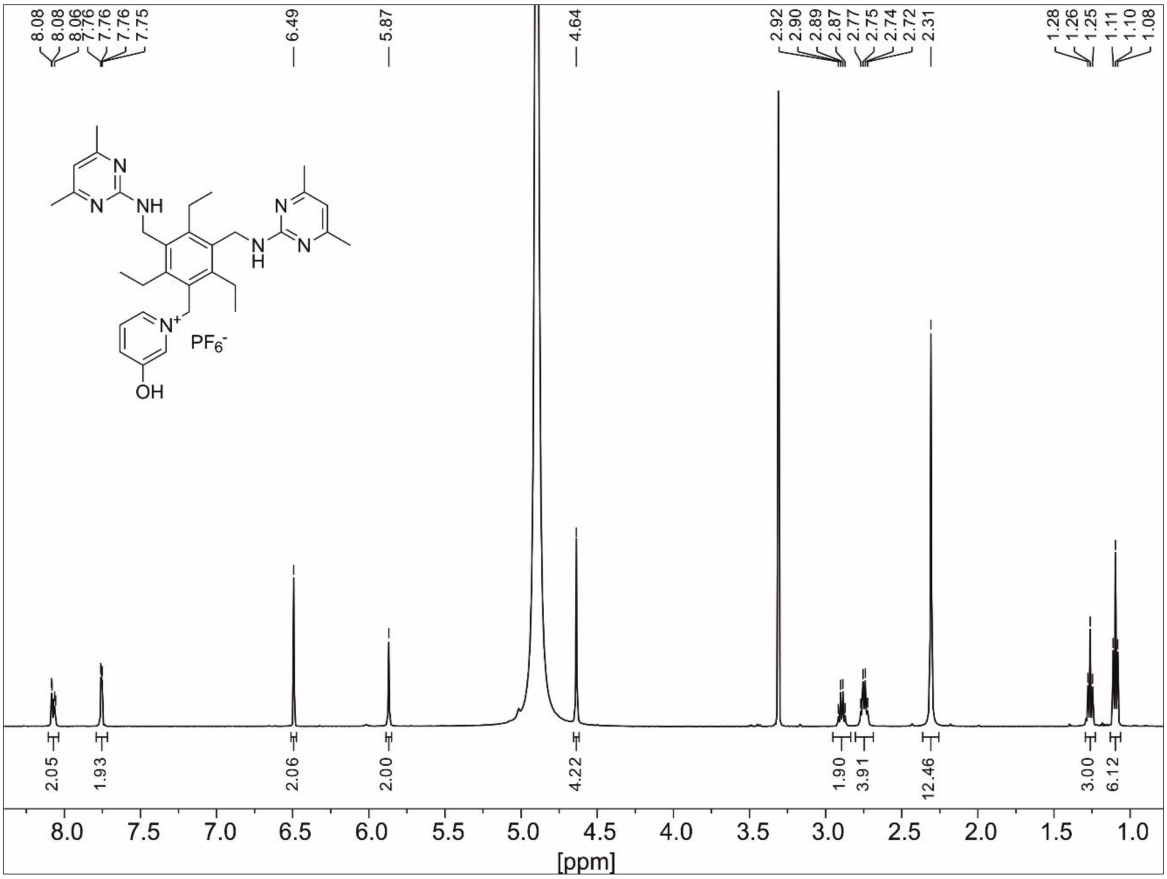

Figure S7a. ^{1}H NMR (500 MHz) spectrum of **3a** in CD_3OD.

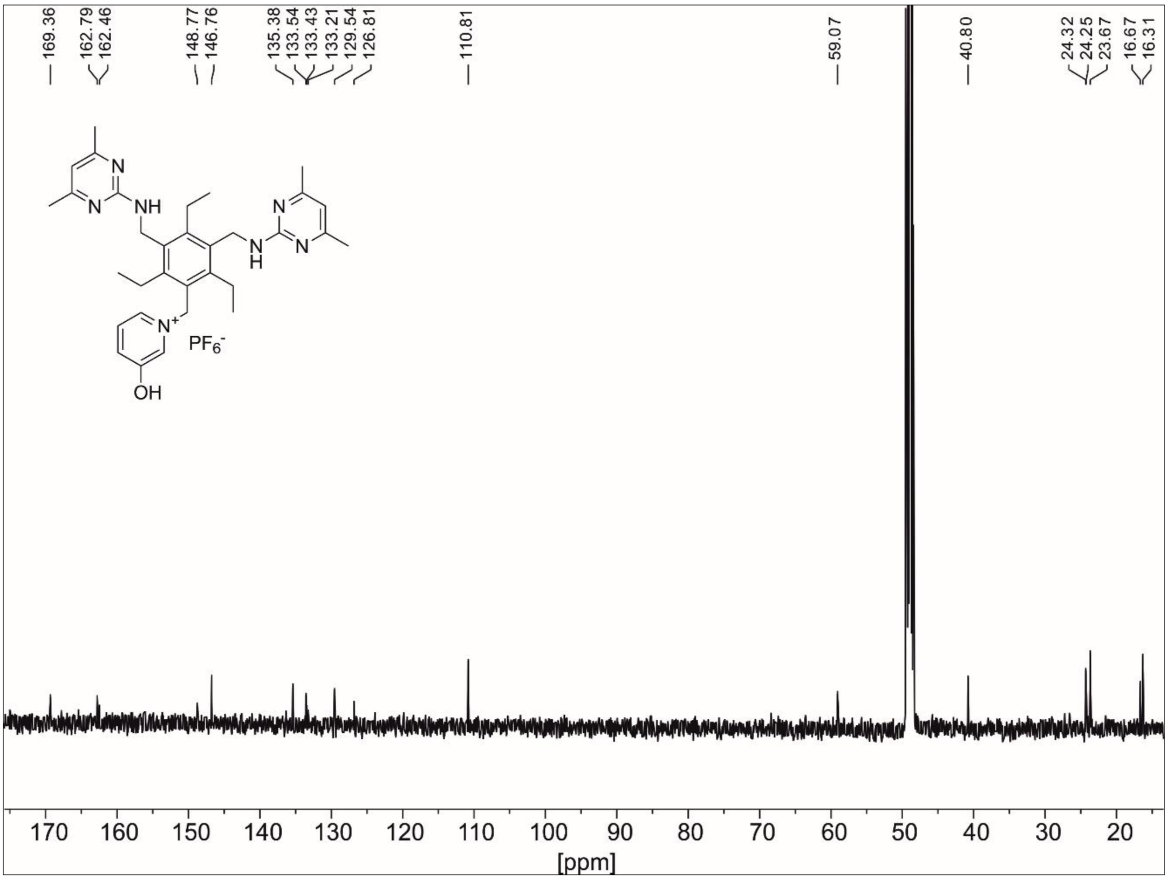

Figure S7b. ^{13}C NMR (125 MHz) spectrum of **3a** in CD_3OD.

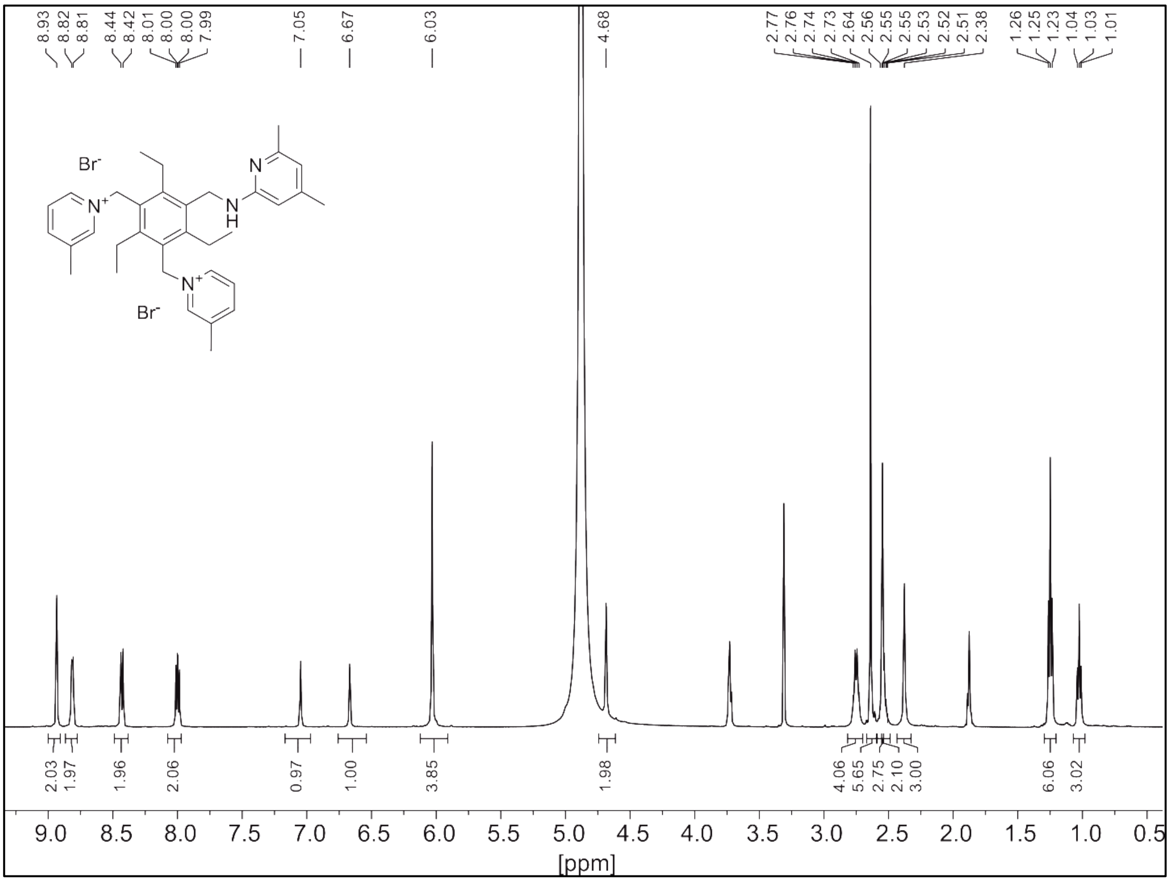

Figure S8a. ^{1}H NMR (500 MHz) spectrum of **4b** in CD_3OD.

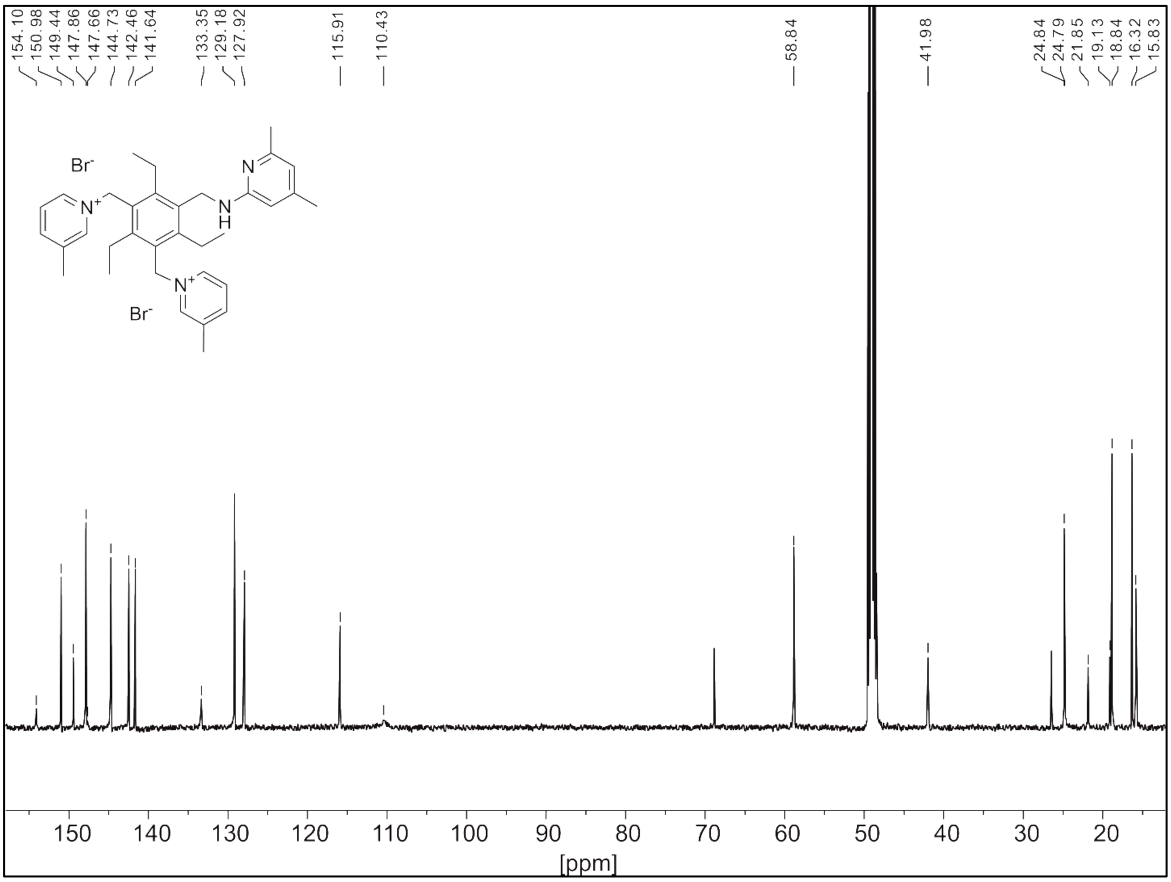

Figure S8b. ^{13}C NMR (125 MHz) spectrum of **4b** in CD_3OD.

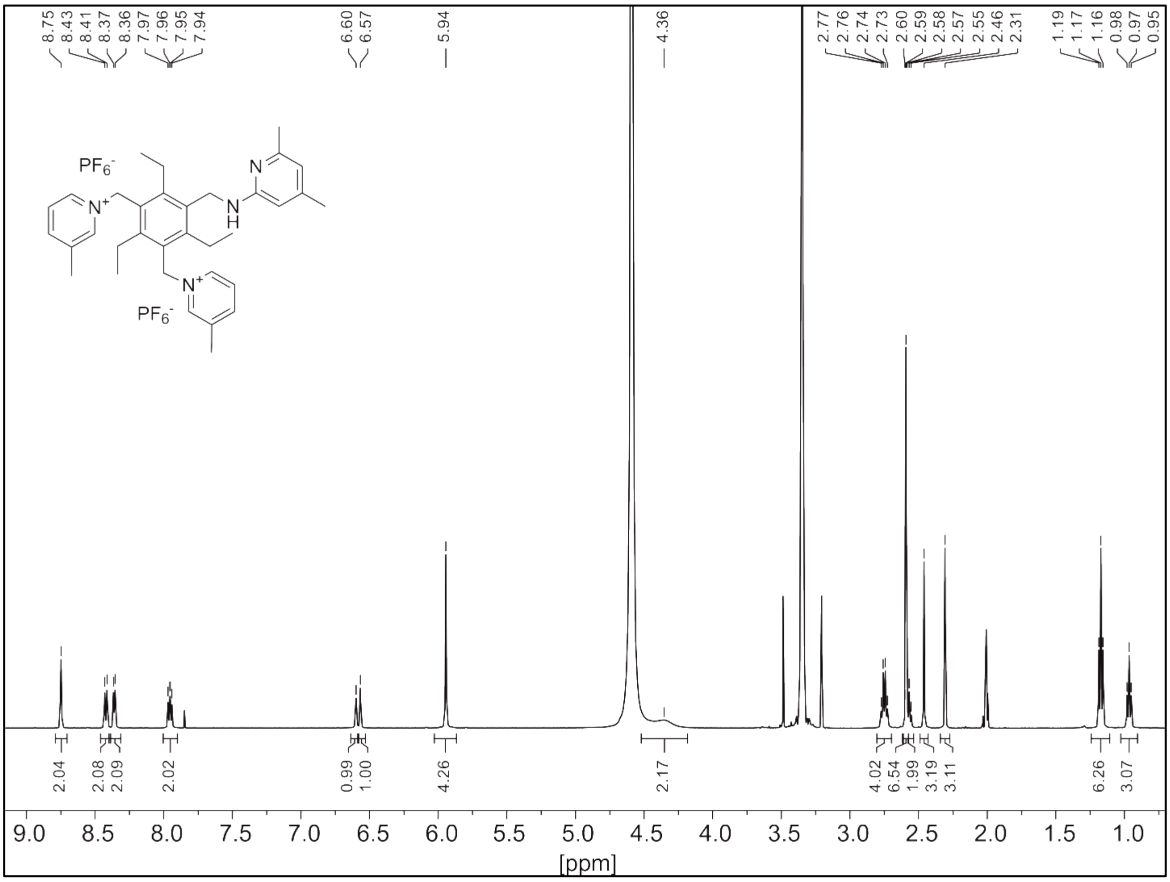

Figure S9a. ^{1}H NMR (500 MHz) spectrum of **4a** in CD_3OD/CD_3CN (2:1, v/v).

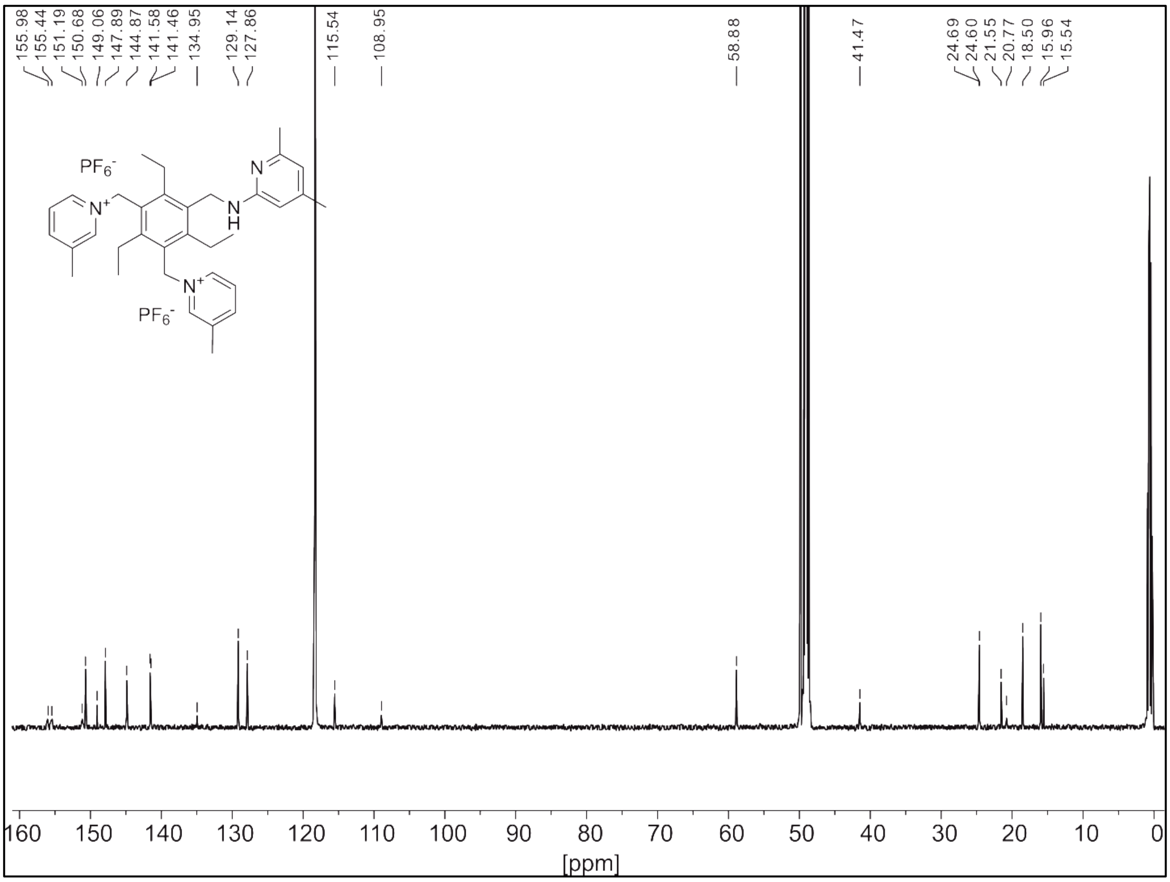

Figure S9b. ^{13}C NMR (125 MHz) spectrum of **4a** in CD_3OD/CD_3CN (2:1, v/v).

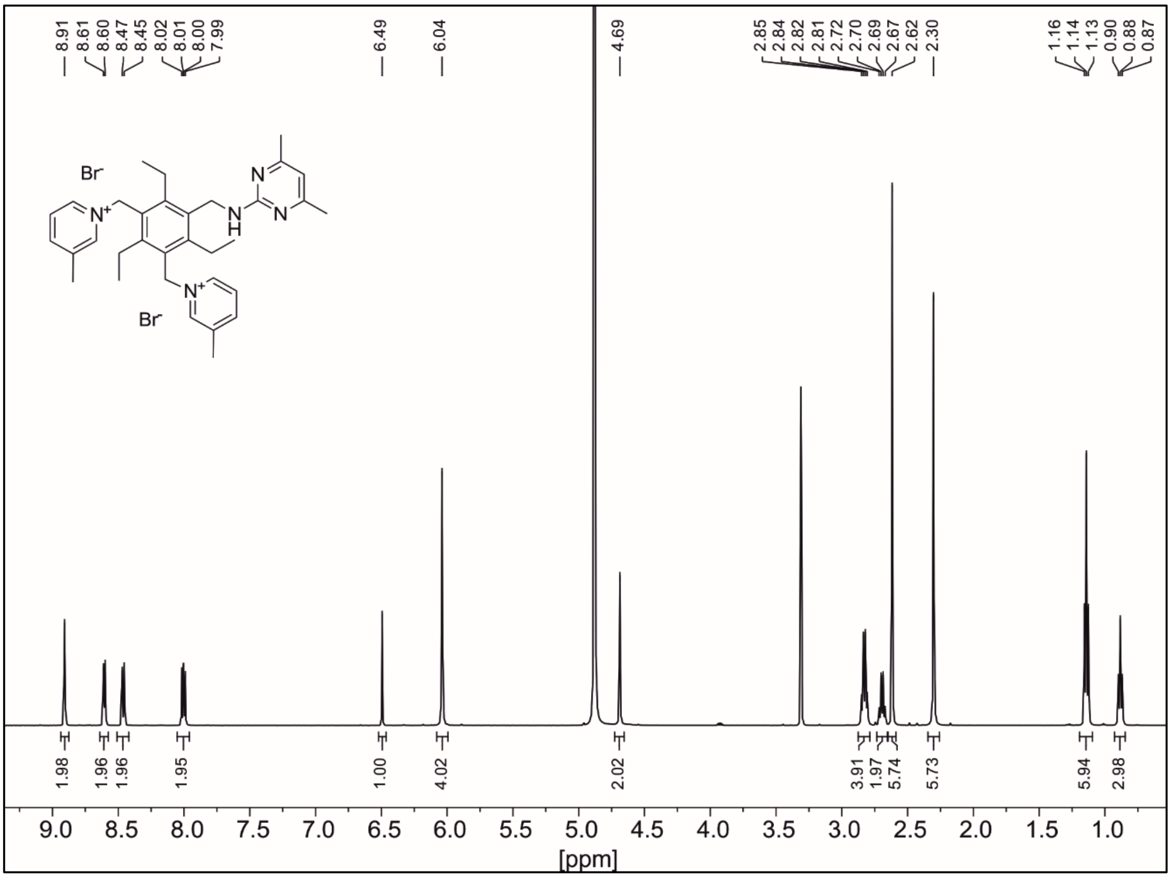

Figure S10a. ^{1}H NMR (500 MHz) spectrum of **5b** in CD_3OD.

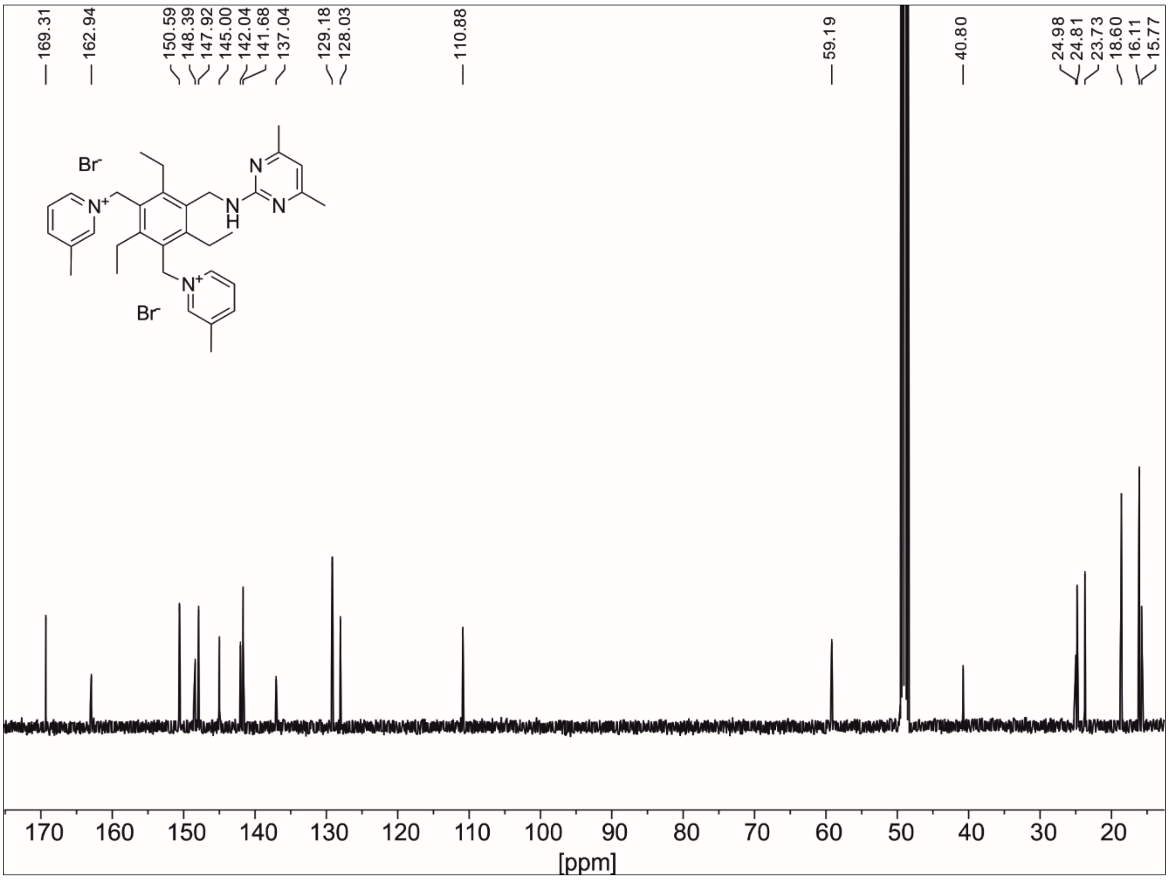

Figure S10b. ^{13}C NMR (125 MHz) spectrum of **5b** in CD_3OD.

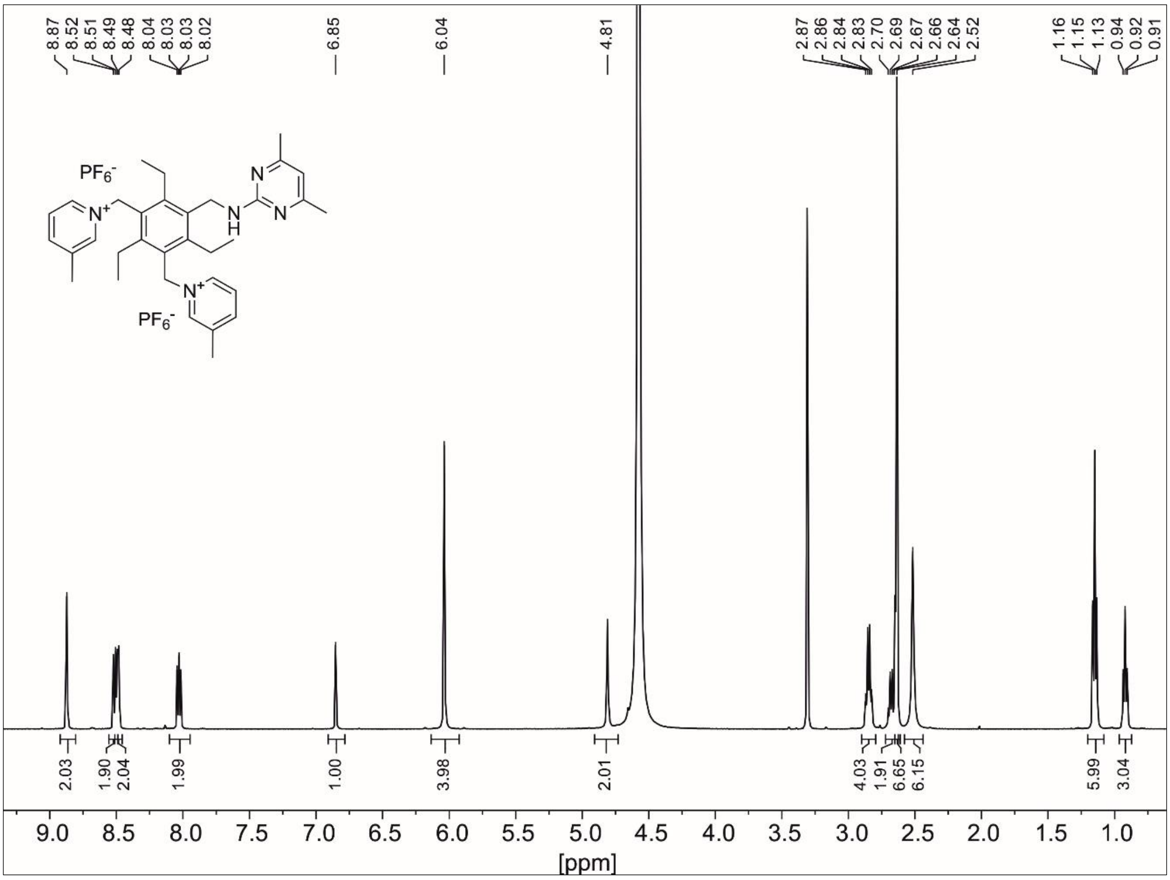

Figure S11a. ^{1}H NMR (500 MHz) spectrum of **5a** in CD_3OD/DMSO-d_6 (5:2, v/v).

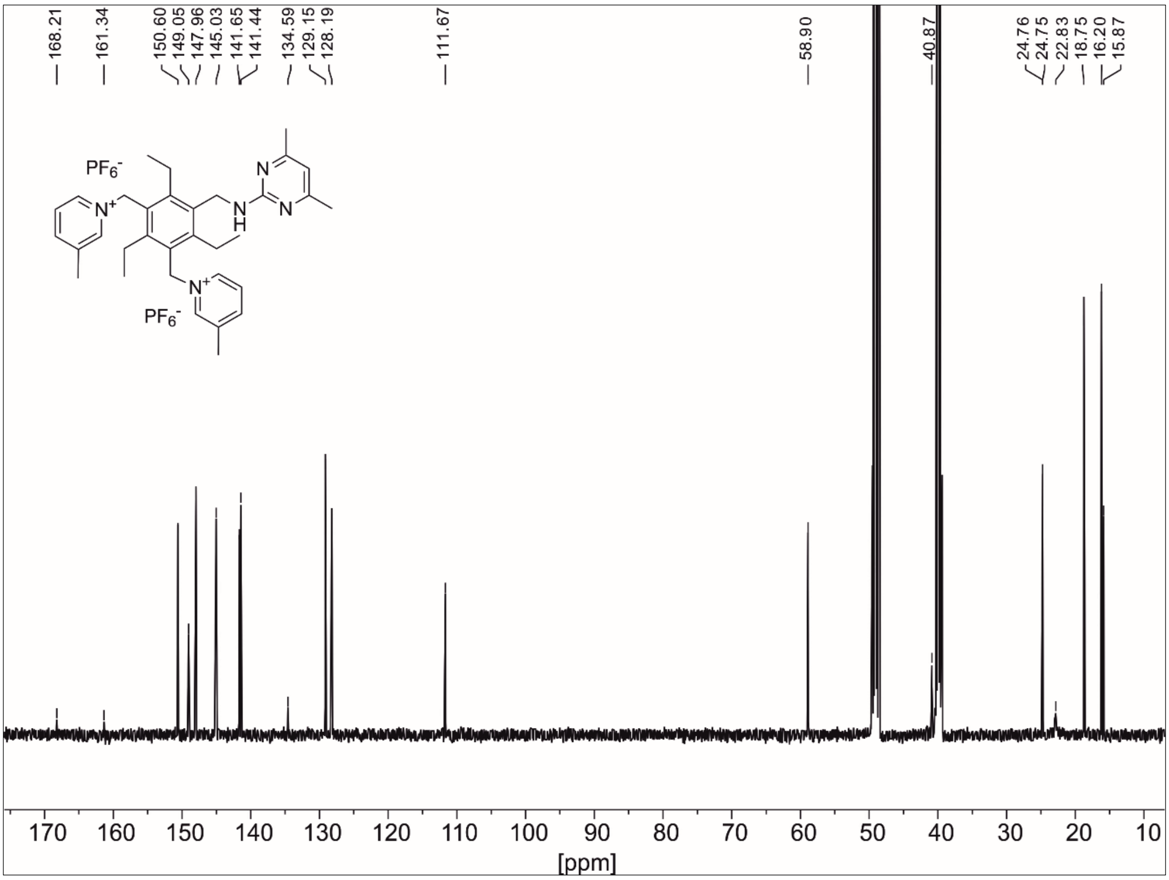

Figure S11b. ^{13}C NMR (125 MHz) spectrum of **5a** in CD_3OD/DMSO-d_6 (5:2, v/v).

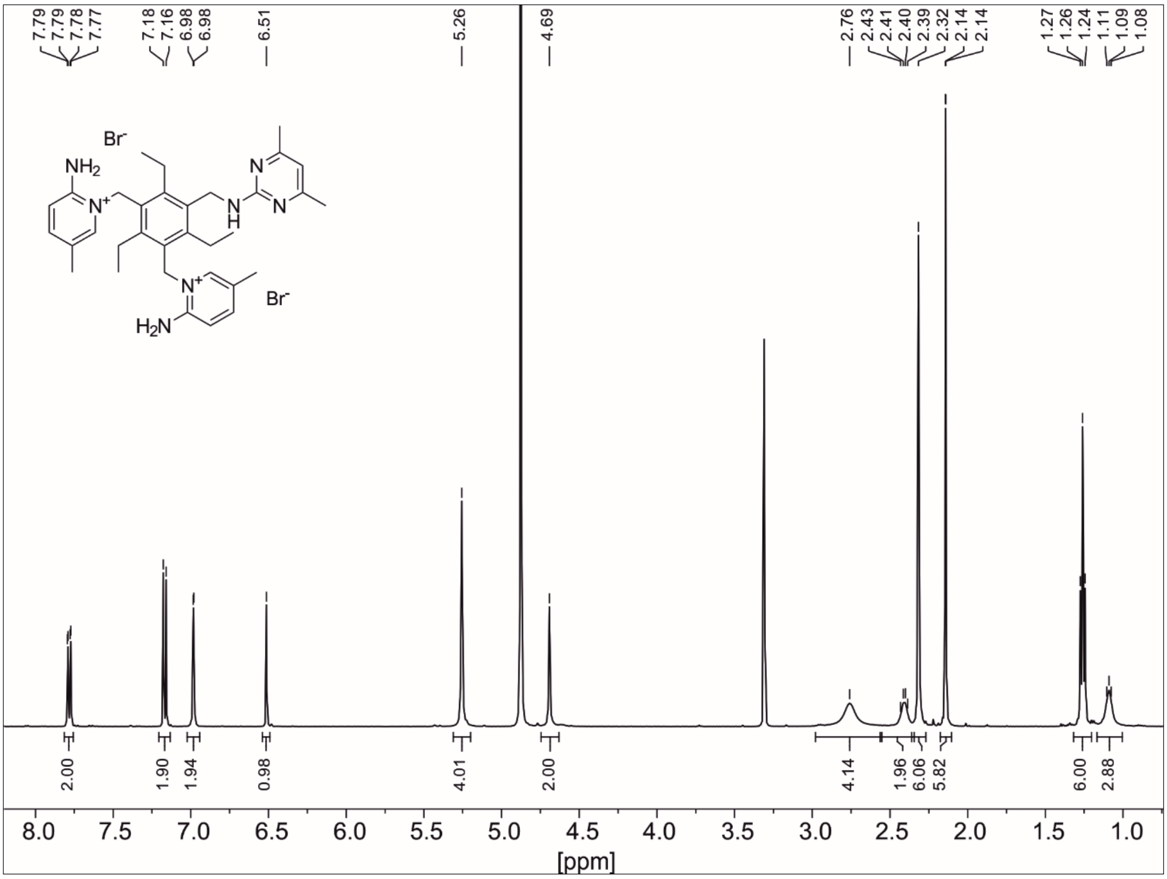

Figure S12a. ^{1}H NMR (500 MHz) spectrum of **6b** in CD_3OD.

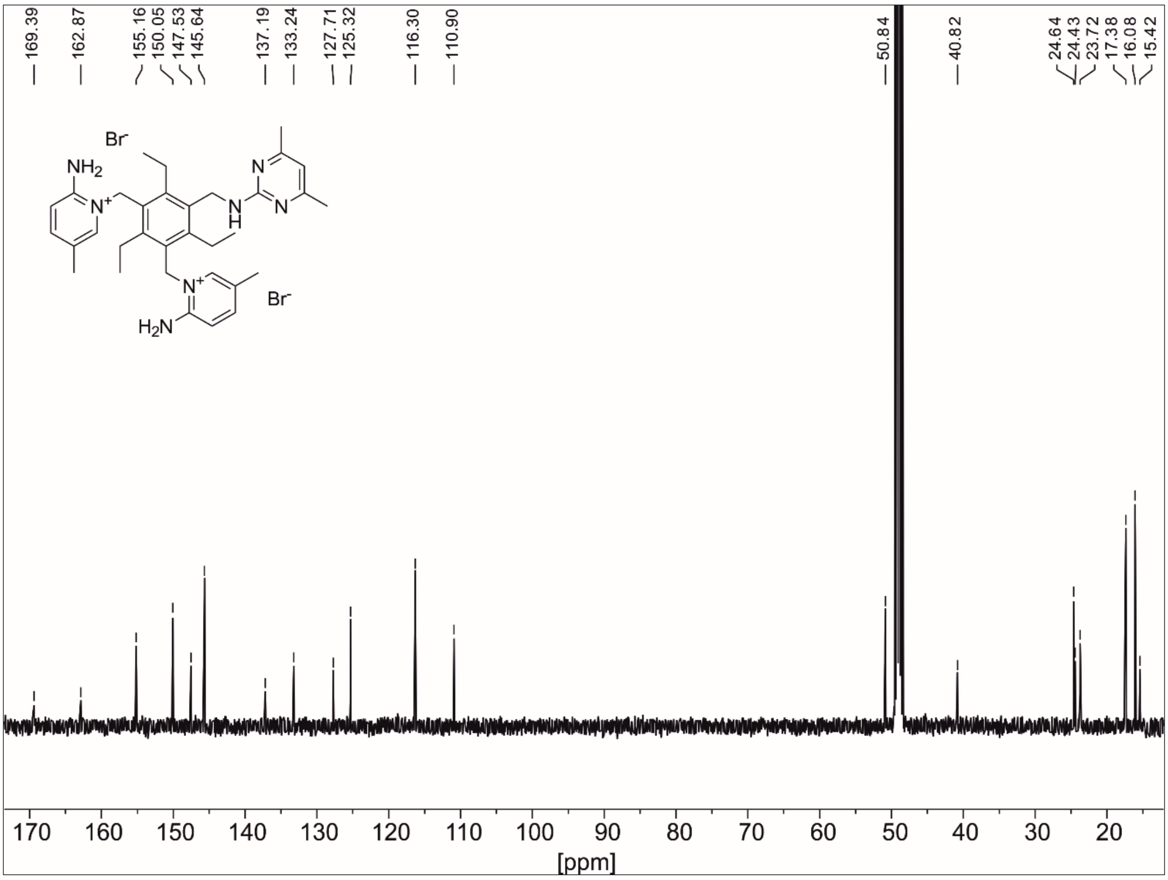

Figure S12b. ^{13}C NMR (125 MHz) spectrum of **6b** in CD_3OD.

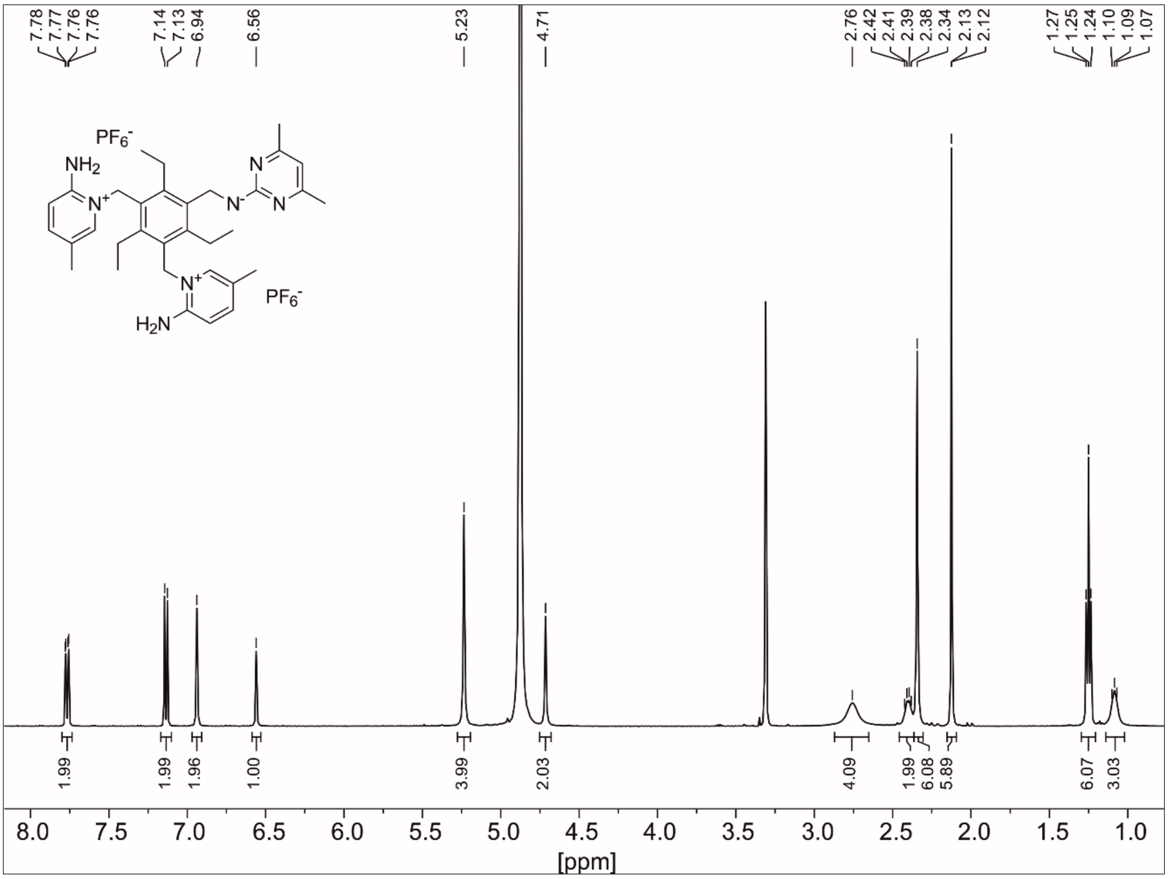

Figure S13a. ^{1}H NMR (500 MHz) spectrum of **6a** in CD_3OD.

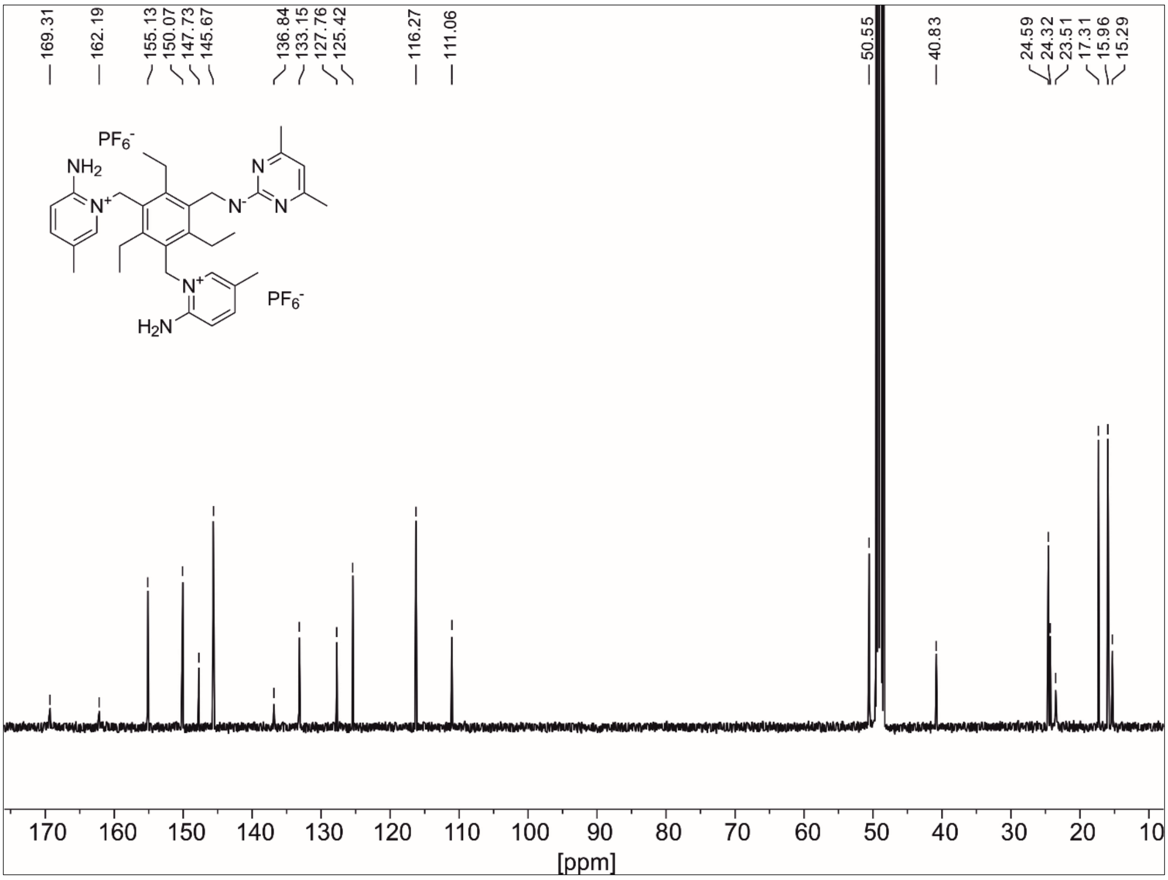

Figure S13b. ^{13}C NMR (125 MHz) spectrum of **6a** in CD_3OD.
